# Prognostic Value of Pre-Operative Transthoracic Echocardiography in Patients with Primary Mitral Regurgitation

**DOI:** 10.31083/j.rcm2511414

**Published:** 2024-11-21

**Authors:** Yun Yang, Lingyun Fang, Wenqian Wu, He Li, Lin He, Manwei Liu, Li Zhang, Yali Yang, Qing Lv, Yuman Li, Jing Wang, Mingxing Xie

**Affiliations:** ^1^Department of Ultrasound Medicine, Union Hospital, Tongji Medical College, Huazhong University of Science and Technology, 430022 Wuhan, Hubei, China; ^2^Clinical Research Center for Medical Imaging in Hubei Province, 430022 Wuhan, Hubei, China; ^3^Hubei Province Key Laboratory of Molecular Imaging, 430022 Wuhan, Hubei, China

**Keywords:** echocardiology, primary mitral regurgitation, surgery, prognosis

## Abstract

Mitral regurgitation is the second most prevalent valvular disease, with primary mitral regurgitation (PMR) accounting for 61%–67% of cases. Chronic PMR can result in progressive left ventricular remodeling and dysfunction, ultimately leading to heart failure or other adverse cardiac events. This, in turn, necessitates frequent referrals, hospitalizations, and cardiac surgeries. The optimal timing for PMR surgery has been a subject of ongoing debate and remains a controversial issue. Presently, it is recommended that patients with chronic PMR undergo earlier mitral valve surgery to enhance post-operative outcomes. For example, the recommendation of European and American guidelines about left ventricular end-systolic diameter for surgery has been altered from 45 mm to 40 mm. Echocardiographic parameters are regarded as noteworthy indicators for intervention in patients with PMR. Extensive research has been undertaken in the field of echocardiography to identify more effective indicators that can propose the optimal timing for surgery, encompassing both conventional and novel echocardiography parameters. However, some parameters are not known to clinicians and the cut-off values for these parameters have shown some variations. Furthermore, a comprehensive review of this topic is currently missing. Consequently, this review aims to provide a thorough summary and elucidation of the prognostic significance of various echocardiographic measurements and their corresponding cut-off values, to help the clinical decision-making and further improve the outcomes of patients with PMR.

## 1. Introduction

Mitral regurgitation (MR) is the second most prevalent valvular disease [1, 2], with primary mitral regurgitation (PMR) accounting for 61%–67% [1, 2]. Chronic PMR can result in progressive left ventricular (LV) remodeling and dysfunction, ultimately leading to heart failure or other adverse cardiac events [3]. This, in return, necessitates frequent referrals, hospitalizations, and cardiac surgery [4]. Besides, the burden of PMR is expected to rise with population aging [5]. The optimal timing for PMR surgery has been a subject of ongoing debate and remains a contentious issue. Presently, it is recommended that patients with chronic PMR undergo earlier mitral valve surgery to enhance post-operative outcomes. For example, the patients would be better off undergoing surgery when their left ventricular end-systolic diameter (LVESD) reaches 40 mm rather than waiting until 45 mm [6, 7, 8]. Current guidelines recommend the presentation of symptoms and left ventricular dysfunction as Class I indications for intervention in patients with PMR [7, 9]. However, left ventricular ejection fraction (LVEF) and LVESD are dependent on LV geometry, heart rate, and volume status [10], and can overestimate the LV systolic function in patients with PMR, although the value of these parameters has been suggested [11, 12, 13, 14, 15, 16]. As a result, more precise and sensitive measures for determining optimal intervention timing are needed.

Furthermore, extensive research has been undertaken in the field of echocardiology to identify more effective indicators that can propose the optimal timing for surgery, encompassing both conventional and novel echocardiographic parameters. However, the question lies in that substantial new parameters and cut-off values were not used in clinical practice, because some variations existed in the published trials and no review has analyzed those results systematically, making it hard to impact decision making.

**Fig. 1.**
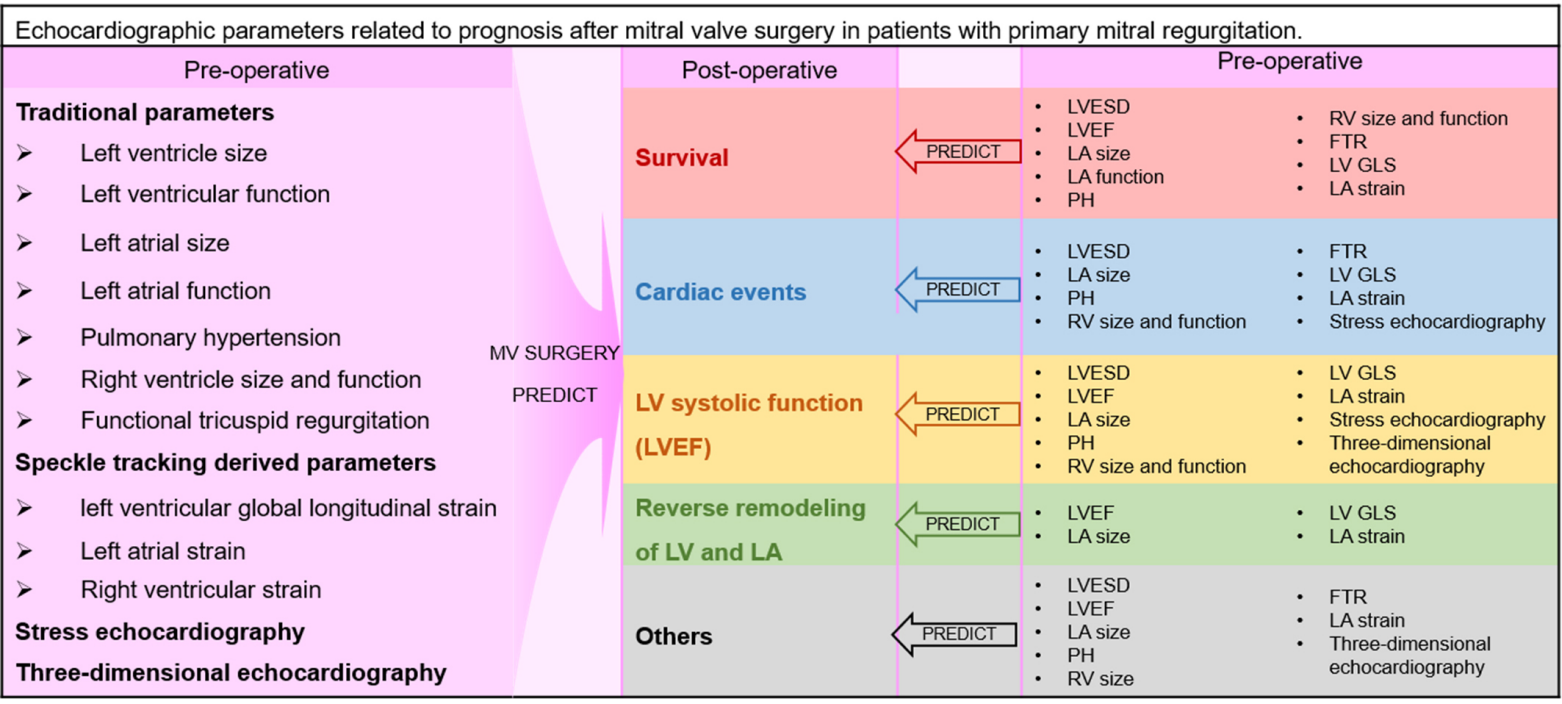
**Echocardiographic parameters related to prognosis after mitral valve surgery in patients with primary mitral regurgitation**. Please note that colors from Fig. 1 are used in all supplementary tables, and different colors are used to distinguish particular post-operative prognosis (red, survival; blue, cardiac events; gold, LVEF; green, reverse remodeling of LV and LA; grey, other post-operative outcomes). LVEF, left ventricular ejection fraction; LV, left ventricle; LA, left atrium; LVESD, left ventricular end-systolic diameter; PH, pulmonary hypertension; FTR, functional tricuspid regurgitation; LV GLS, left ventricular global longitudinal strain; RV, right ventricle; MV, mitral valve.

Consequently, in order to help the clinical decision-making and further improve the outcomes of patients with PMR, this review aims to provide a comprehensive summary and elucidation of the published evidences concerning the prognostic significance of these parameters and their cut-off values (Fig. 1).

## 2. Traditional Parameters

### 2.1 Left Ventricle Size

LVESD was a well-recognized prognostic parameter for post-operative LV performance in patients who underwent mitral valve (MV) surgery [17]. It is demonstrated that pre-operative LVESD was useful for predicting various outcomes, including LV dysfunction [18, 19, 20, 21, 22, 23], LVEF deterioration [14], cardiac events [23], as well as mortality [15, 16] during follow-up (**Supplementary Table 1**).

Based on previous studies of the predictive value of LVESD [15, 20, 21], American College of Cardiology/American Heart Association (ACC/AHA) have recognized LVESD ≥40 mm as a Class I indication for MV surgery since 2006 [8], while the European Society of Cardiology and the European Association for Cardio-Thoracic Surgery (ESC/EACTS) updated their recommendation from 45 mm [6] to 40 mm in 2021 [7], to achieve better short- and long-term outcomes.

However, some studies have reported different cut-off values for LVESD. For instance, Tribouilloy *et al*. [22] found a cut-off value of 37 mm to predict LV dysfunction, while Kitai *et al*. [19] identified 39 mm as a predictor for LV dysfunction. These values differ from the guideline-recommended 40 mm. Additionally, Li *et al*. [14] discovered that in patients with PMR and LV systolic dysfunction (LVEF <60% or LVESD >40 mm), pre-operative LVESD ≥45 mm was independently associated with much worsened LV systolic function 3.1 ± 2.6 years after surgery.

Other parameters reflecting LV size have also been studied for their predictive value. Left ventricular end-systolic diameter index was demonstrated to be predictive of LV dysfunction six months after surgery, with a cut-off value of 22 mm/m^2^ [21]. Left ventricular end-systolic volume index (LVESVi) was identified as the best marker of LV impairment one year after operation, with an optimal cut-off of 36.3 mL/m^2^ (area under curve (AUC) 0.738 [0.56–0.71]) [24]. Furthermore, left ventricular end-diastolic diameter (>60 mm) can predict recurrent MR 3.8 ± 2.7 years after surgery (hazard ratio (HR) 1.88 [1.06–3.34], *p* = 0.03) [16].

### 2.2 Left Ventricular Function

LVEF is a classical parameter to assess LV systolic function. It has been demonstrated that pre-operative LVEF holds prognostic value in predicting post-operative LV dysfunction [13, 19, 22, 23, 25, 26], a reduction in LVEF [14], left atrial reverse remodeling (LARR) [27], recurrent MR [16], and mortality [12, 16] during follow up (**Supplementary Table 2**). This indicates the importance of assessing LVEF prior to surgery as it can provide valuable insights into the potential outcomes and complications that may arise after the procedure.

Based on previous studies [25, 26], LVEF <60% is regarded as a Class I indication for intervention for chronic PMR by both ACC/AHA and ESC/EACTS recommendations [7, 9].

However, the cut-off values of LVEF reported by some studies are different, such as 63% suggested by Kitai *et al*. [19] and 64% suggested by Tribouilloy *et al*. [22]. In addition, for PMR patients with LVEF <60% or LVESD >40 mm, pre-operative LVEF ≤52% was an independent risk factor for post-operative worsened LV systolic function [14]. Relatively, for patients with LVEF >60% and LVESD <40 mm, pre-operative LVEF ≤65% was identified as the optimal cut-off value to predict early post-operative LV dysfunction [13]. Except for LVEF, there are some other indices reflecting LV systolic function that have the potential to predict post-operative outcomes. It is indicated that forward LV stroke volume and forward LVEF can be predictive of prolonged intensive care unit (ICU) stay [28] and occurrence of composite events [11]. Mitral valve velocity (S’) was also reported to be predictive of post-operative LV reverse remodeling [29] and post-operative LVEF reduction >10% [30].

LV Tei index (myocardial performance index) serves as a measure of both systolic and diastolic function, but its predictive value has shown inconsistency across various studies. In a study by Mabrouk-Zerguini *et al*. [31] involving 25 patients who underwent MV repair, the Tei index was found to be capable of predicting immediate post-operative LV fractional area change. This predictive ability was noted to assist in identifying patients who might encounter challenges during the weaning process from cardiopulmonary bypass. However, a study by Mukherjee *et al*. [32], which included 130 patients undergoing MV repair, revealed that pre-operative Tei index was not able to predict left ventricular function immediately after surgery.

While the predictive value of diastolic function in PMR has been explored, the existing research is limited. One study reported that late diastolic tissue Doppler imaging a^′^ (a^′^-TDI) wave of the septal apical wall (<3.2 cm/s, AUC 0.82) and the mid-septal wall (<3.66 cm/s, AUC 0.82) emerge as the most significant predictive factors for post-operative recurrent MR [33]. Additionally, the early diastolic tissue Doppler imaging e^′^ (e^′^-TDI) wave was found to be associated with a reduction in LA maximum volume and an increase in active atrial emptying fraction [34]. Nevertheless, further investigations into diastolic function are warranted to deepen our understanding in this area.

### 2.3 Left Atrial Size

Left atrial (LA) size plays a crucial role in patients with chronic PMR because LA can adapt to the volume overload resulting from regurgitation [35]. LA size can be measured by LA dimension, area, and volume [36, 37]. Many related studies showed associations between pre-operative enlarged LA and post-operative adverse outcomes [13, 37, 38, 39, 40, 41, 42, 43], such as atrial fibrillation (AF) [40, 43], all-cause mortality [37, 38, 39], cardiac events [39] and LV dysfunction [13] (**Supplementary Table 3**). The current ESC/EACTS guideline recommends that enlarged LA (volume index ≥60 mL/m^2^ or diameter ≥55 mm) as Class IIa indication for intervention of PMR [7]. What’s more, most studies found larger left atrial volume index (LAVi) was independently associated with more significant post-operative LARR [40, 41, 42, 43], despite their post-operative LA size level remaining the highest (worst) [43].

However, Song *et al*. [44] showed that pre-operative LAVi was negatively associated with post-operative LARR (β = –0.595, *p* = 0.001). The discordant result of Song *et al*.’s study [44] could partially be explained by its highest baseline LAVi ([58.2 ± 15.7] mL/m^2^). Larger LA size is implicated with AF through increased stretch and interstitial fibrosis, which is difficult to recover [45]. Moreover, Hu *et al*. [13] identified pre-operative LAVi 53 mL/m^2^ as the optimal cut-off value to predict early post-operative LV dysfunction after studying 623 asymptomatic patients with LVEF >60% and LVESD <40 mm. What’s more, despite the predictive significance of LA size being supported by a substantial body of research, the independence of LAVi as a risk factor was still doubted [46]. The poor prognosis carried by enlarged LA size could be implicated with MR severity and duration, larger LV size, overt or masked LV systolic or diastolic dysfunction, AF, pulmonary hypertension (PH), tricuspid regurgitation (TR), or right-sided heart dysfunction, making the mechanisms underlying such adverse outcomes not so clear and needing further research.

### 2.4 Left Atrial Function

Except for emerging data about the prognostic value of LA volume in PMR, LA function assessment has also started to be concerned [47]. LA coupling index is a clinically achievable parameter that couples LA volumetric and mechanical characteristics, calculated as the ratio of LAVi to a^′^-TDI at the mitral annulus. Through analysis of a large cohort of PMR patients, Essayagh *et al*. [48] demonstrated that higher LA coupling index was associated with higher mortality (HR 1.13 [1.00–1.24], *p* = 0.04 per 3 units), and LA coupling index has significant incremental predictive power over LAVi (*p* < 0.0001) (**Supplementary Table 3**).

AF occurs around in 30–42% of PMR patients at diagnosis [45, 49, 50]. AF was confirmed to be an independent predictive factor of worse outcomes, including excess long-term mortality [50, 51] and post-operative LV dysfunction [19, 20, 45, 49, 50], although AF is associated with older age, severity of PMR and LA size [49, 50]. AF is considered a prompt trigger for intervention regardless of patients’ symptoms [19, 20, 45, 50].

### 2.5 Pulmonary Hypertension

A series of downstream changes can occur following chronic PMR. Volume overload caused by PMR leads to a sustained increase in LA volume and pressure. Consequently, it is transmitted backward into the pulmonary veins and then PH occurs [52]. Then the elevated right ventricular (RV) afterload can lead to right heart remodeling, functional tricuspid regurgitation (FTR), and finally RV dysfunction or failure [52]. The interactions among PH, RV size and function, and FTR remain complex and their prognostic value have been studied.

It is reported that approximately 20%–30% of patients with severe PMR were present with significant PH (systolic pulmonary arterial pressure [SPAP] ≥50 mm Hg) [53, 54]. Previous studies demonstrated that PH could be a marker for poor post-operative outcomes including in-hospital mortality or death within 30 days of operation [55], late all-cause mortality [51, 53, 54, 55, 56, 57, 58, 59], cardiovascular mortality [53, 54], major adverse cardiac and cerebrovascular events [58], reoperation [51], LV dysfunction [60, 61], and post-operative persistent PH [57] (**Supplementary Table 4**). Furthermore, SPAP was described to have incremental value for post-operative mortality [54, 57]. The latest guidelines regard resting PH as one of Class IIa indications for MV surgery [7, 9].

What’s more, Le Tourneau *et al*. [54] reported that SPAP cut-off value of 50 mmHg could predict all-cause mortality with a sensitivity of 61% and a specificity of 72% (AUC 0.7, *p* < 0.0001) while a cut-off value 45 mmHg with a better sensitivity of 71% but a weaker specificity of 62% (AUC 0.7, *p* < 0.0001). However, the study of Murashita *et al*. [58] did not find any association between pre-operative PH and post-operative 30-day mortality. The difference may partially be explained by its smaller sample size or the different statistical analysis methods used compared with the study of Ghoreishi and colleges [55].

Additionally, although cardiac catheterization is the gold standard for PH diagnosis, echocardiology is the most convenient and commonly used tool to evaluate SPAP in clinical practice, it is worthy paying more attention to the prognostic value of echocardiology-derived PH in patients with PMR.

### 2.6 Right Ventricle Size and Function

Generally, dilated RV size and impaired RV function are often indicative of a more advanced stage of disease progression [52]. The prognostic significance of RV size and function in patients with PMR has been the subject of investigation, with various parameters used to evaluate the RV, including RV size, RV fractional area change (RV FAC), right ventricular ejection fraction (RV EF), tissue Doppler–derived tricuspid lateral annular systolic velocity (s^′^-TDI), tricuspid annular plane systolic excursion (TAPSE), and RV index of myocardial performance (RV MPI) [62].

Studies have shown significant associations between RV parameters and clinical outcomes in patients undergoing MV surgery (**Supplementary Table 5**). For instance, Gackowski *et al*. [28] reported that right ventricular end-diastolic diameter (RVEDD) was independently predictive of a prolonged ICU stay after MV replacement, with the cut-off value being 35 mm. Haddad *et al*. [63] pointed out that a smaller RV FAC was linked to in-hospital mortality or circulatory failure (odds ratio [OR] 0.001 [<0.001–0.727] per 1% increment, *p* = 0.048) in patients undergoing left-sided valve surgery. Le Tourneau and colleagues [64] demonstrated that RV EF of 35% or lower was associated with higher cardiovascular mortality in patients with chronic PMR. Furthermore, Chrustowicz *et al*. [65] reported associations between s^′^-TDI and TAPSE with reductions in LVEF (>10%) post-surgery, with the cut-off values of s^′^-TDI and TAPSE of 8.75 mm/s and 17.5 mm, respectively. Haddad *et al*. [63] found that RV MPI of 0.50 or higher was associated with in-hospital mortality or circulatory failure (OR 25.20 [5.24–121.15]), and could provide incremental predictive value for post-operative adverse outcomes. Ye *et al*. [66] studied 781 patients underwent surgery for PMR, revealing that higher RV MPI was associated with late death. Combining multiple RV parameters may offer a more reliable approach to identifying abnormal RV function due to the complex geometry and structure of the RV [62]. Towheed and associates [67] demonstrated that RV dysfunction, defined as having at least three abnormal RV parameters out of 5 (RV FAC, TAPSE, S’, RV MPI, and RV dP/dt), was a strong predictor of 30-day mortality (OR 3.5 [1.1–11.1], *p* = 0.03) and composite adverse events (OR 4.2 [2.1–8.3], *p* < 0.01) after left-sided valve surgery. This underscores the importance of comprehensive RV assessment in predicting outcomes in patients with PMR undergoing cardiac surgery.

### 2.7 Functional Tricuspid Regurgitation

FTR is not uncommon in patients requiring MV surgery, with the prevalence ranging from 25% to 59% [68, 69], and the prevalence of moderate or greater FTR ranging from 8% to 45% [70, 71]. The studies concerning the effects of FTR were summarized in **Supplementary Table 6**. It is demonstrated that increasing grades of pre-operative FTR was associated with poorer post-operative survival [69, 70, 72, 73, 74], congestive heart failure [69, 70] or FTR progression [69] during long-term follow up. Given these poor prognoses associated with FTR, concomitant surgical or transcatheter treatments of FTR have been more frequent in recent years [75]. Current ESC/EACTS and ACC/AHA guidelines recommended that, for FTR attributed to left-sided valve diseases, tricuspid valve surgery be performed in patients with severe FTR undergoing concomitant left-sided valve surgery (Class I) [9], or in patients with progressive FTR and tricuspid annulus end-diastolic diameter >40 mm or signs and symptoms of right heart failure undergoing concomitant left-sided valve surgery (Class IIa) [9], or isolated tricuspid valve surgery in patients with severe FTR because of annular dilation and signs and symptoms of right heart failure (IIa) [7, 9]. However, controversies still existed in patients with mild or moderate FTR regarding surgical time, mortality, morbidity, congestive heart failure, and FTR progression post-operatively [73, 76, 77, 78, 79].

The benefits and risks of tricuspid valve surgery in patients with mild or moderate FTR need to be further investigated and considered.

## 3. Speckle Tracking-Derived Parameters

### 3.1 Left Ventricular Global Longitudinal Strain

Most studies consistently indicate that impaired pre-operative left ventricular global longitudinal strain (LV GLS) serves as a prognostic indicator for adverse outcomes in patients with PMR [18, 20, 21, 24, 30, 39, 80, 81, 82, 83, 84, 85, 86] as shown in **Supplementary Table 7**. The cut-off values for LV GLS predicting worse outcomes typically ranged from –17.9% to –20.5%. Additionally, the incremental value of LV GLS over traditional parameters has been underscored [20, 39, 80, 83, 84, 87]. However, there are some divergent findings in the literature. For instance, Pandis *et al*. [87] noted that an LV GLS better than –20.5% was associated with a greater reduction of LVEF, and an LV GLS better than –17.9% was associated with LVEF reduction of more than 10% and resultant LVEF below 50%. Song *et al*. [23] did not find any association between pre-operative LV GLS and LV dysfunction at 1 week and 3 months post-operation possibly due to unstable hemodynamics immediately following surgery and the inclusion of a heterogeneous patient population.

Twist, another parameter derived from speckle tracking echocardiography, has also been investigated. Candan *et al*. [18] examined 59 asymptomatic severe MR patients who underwent surgery and discovered that pre-operative twist was associated with post-operative LVEF (r = 0.42, *p* = 0.001) and independently predicted post-operative LV function (OR 0.8 [0.64–0.96], *p* = 0.02).

In summary, impaired pre-operative LV GLS could significantly predict negative outcomes but the cut-off values varied within a relatively narrow range because of differences in vendor-specific software.

### 3.2 Left Atrial Strain

LA strain indices are of increasing value to assess LA function [88]. Studies have shown that decreased pre-operative LA reservoir strain was one of the independent and incremental predictors of poorer outcomes after MV surgery [42, 84, 85, 89, 90] in terms of mortality, cardiac events, LV dysfunction, and functional capacity (**Supplementary Table 8**). An LA reservoir strain worse than cut-off value 21% may predict higher risk of post-operative cardiac events [90]. An LA reservoir strain worse than 22% could predict long-term post-operative all-cause mortality [85]. Besides, Cameli *et al*. [91] reports that LA strain impairment may appear earlier than LV strain because LA is the most susceptible chamber due to its’ thinner wall and MR-induced volume overload. In the future, studies about LA strain should take the thin walls of LA and the lack of normal values across various vendor-specific software into account [92].

### 3.3 Right Ventricular Strain

Study concerning the predictive value of RV longitudinal strain in patients after MV surgery for PMR were scarce. Kislitsina *et al*. [84] reported that worse RV free wall longitudinal strain was associated with post-operative LV dysfunction (OR 1.171 [1.015–1.351]), and incremental prognostic value of strain (LV GLS, RV free wall longitudinal strain, LA reservoir strain) on top of demographics and other echocardiography parameters was demonstrated, but on multivariable Cox survival analysis, RV free wall longitudinal strain was not shown to be significantly associated with mortality (HR 1.078 [0.952–1.231], *p* = 0.243). More investigations about the predictive value of RV dysfunction in patients with PMR are needed.

## 4. Stress Echocardiography

Stress echocardiography has emerged as a valuable tool for identifying latent ventricular dysfunction [7, 9], which may present as deteriorated echocardiographic measurements or limited contractile reserve in pharmaceutical or exercise stress echocardiography [86, 93, 94, 95, 96]. Concerning PMR, stress echocardiography plays a crucial role in guiding clinical decisions for patients with discrepancies in regurgitation severity, symptoms, or LV function at rest [7, 9].

Studies investigating the prognostic utility of stress echocardiography have highlighted several key findings, as outlined in **Supplementary Table 9**. Exercise-induced parameters such as LVEF (with a cut-off value of 68%) [96], LVESVi (cut-off value 25 cm^3^/m^2^) [96], and LV GLS normalized for LVESD (cut-off value –5.7%/cm) [86, 95] have been identified as predictors of post-operative LVEF. Additionally, exercise-induced PH (SPAP >60 mmHg), TAPSE (cut-off value 26 mm), mitral early filling-wave velocity/e’-TDI (E/e’), effective regurgitant orifice area and positive exercise stress echocardiography could predict major adverse cardiovascular events following surgery [93, 94, 97, 98]. Besides, Lee *et al*. [99] reported that the contractile reserve of LVEF (cut-off value 4%) and LV GLS (cut-off value 1.9%) [86, 95] were associated with post-operative LVEF.

In conclusion, exercise echocardiography offers valuable insights in PMR cases where resting two-dimensional echocardiography results are inconclusive or ambiguous. However, despite its clinical significance, the widespread adoption of exercise echocardiography in PMR patients is hindered by its time-consuming and labor-intensive nature. Further research and advancements are needed to streamline and optimize the clinical application of exercise echocardiography in the management of PMR.

## 5. Three-Dimensional Echocardiography

Significant advancements in three-dimensional (3D) echocardiography have revolutionized the visualization of pathomorphological features of heart valves in real-time [100], as well as the precise quantification of chamber dimensions and regurgitation volumes [101], thereby enhancing its clinical utility in MV disease [102]. When compared with two-dimensional echocardiology, 3D echocardiology allows for more accurate pathomorphological characterization and function evaluation.

Studies have indicated that 3D echocardiography is valuable in predicting post-operative outcomes in patients with PMR. Yingchoncharoen *et al*. [103] found that 3D LVESVi independently predicted the post-operative development of AF or LV dysfunction (OR 1.06 [1.04–1.16], *p* < 0.001) beyond other clinical and echocardiographic parameters. This highlights the incremental prognostic value of 3D LVESVi in post-operative outcomes. Additionally, Tokodi *et al*. [104] demonstrated that 3D RV longitudinal ejection fraction (OR 1.33 [1.08–1.77], *p* < 0.05) and 3D RV GLS were correlated with post-operative RV dysfunction (OR 0.82 [0.68–0.94], *p* < 0.05).

While the scientific literature and clinical applications of 3D echocardiography are expanding, its primary use remains in transesophageal echocardiography. Transthoracic 3D echocardiography is limited by lower frame rates and spatial resolution. Future advancements in technology may address these limitations, potentially enhancing the role of 3D echocardiography in routine clinical practice.

## 6. Summary

**Fig. 2.**
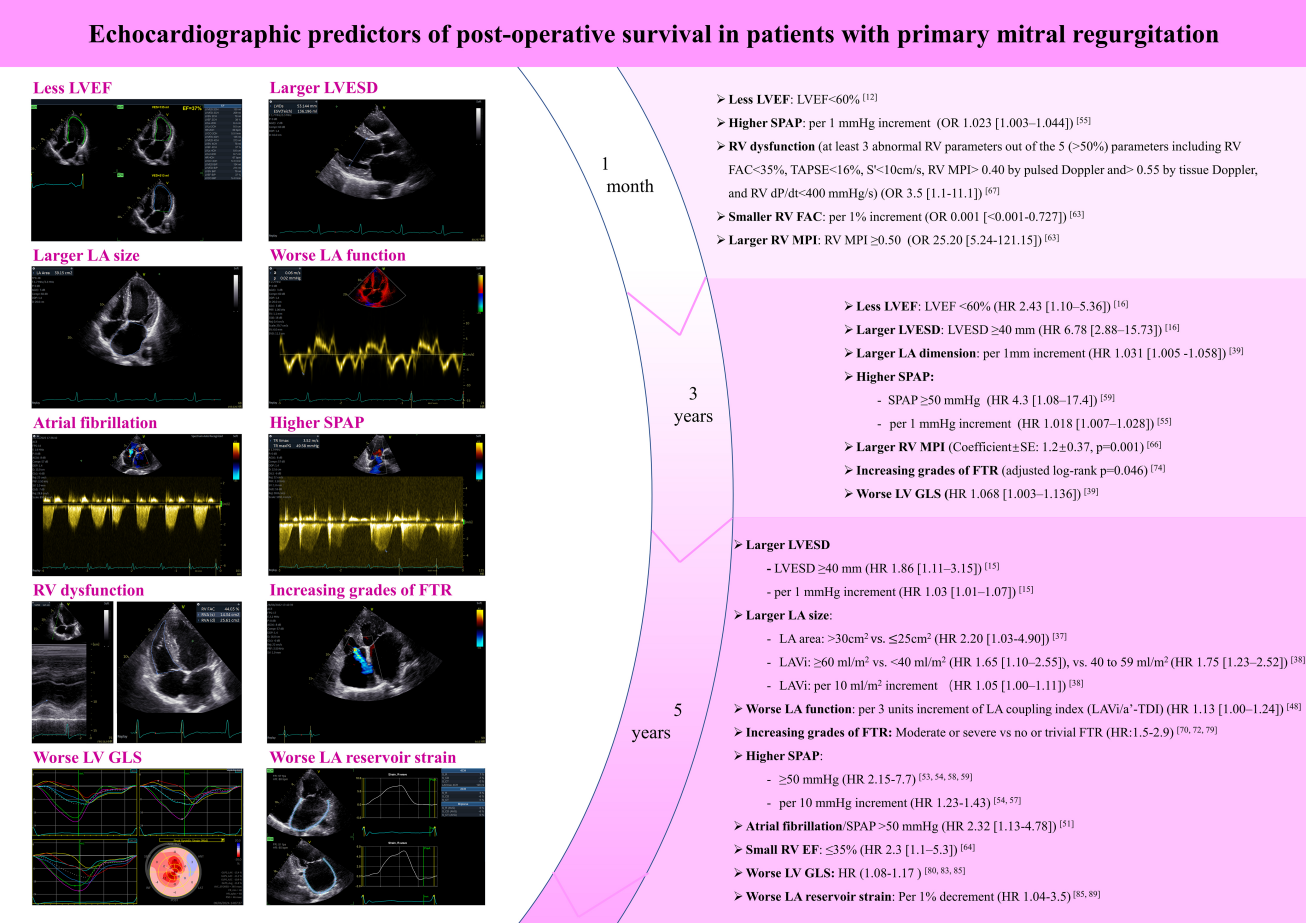
**Echocardiographic predictors of post-operative survival in patients with primary mitral regurgitation**. LVEF, left ventricular ejection fraction; LVESD, left ventricular end-systolic diameter; LA, left atrium; SPAP, systolic pulmonary arterial pressure; RV, right ventricle; FTR, functional tricuspid regurgitation; LV GLS, left ventricular global longitudinal strain; RV FAC, RV fractional area change; RV MPI, RV index of myocardial performance; LAVi, left atrial volume index; RV EF, RV ejection fraction; a^′^-TDI, late diastolic tissue Doppler imaging a^′^; OR, odds ratio; HR, hazard ratio; TAPSE, tricuspid annular plane systolic excursion; CH, chamber; VES,volume in end-systole; VED, volume in end-diastole; EF, ejection fraction; LVVES, left ventricular volume in end-systole; LVVED, left ventricular volume in end-diastole; LVSV, left ventricular stroke volume; LVEF, left ventricular ejection fraction; LVLs, left ventricular length at systole; LVLd, left ventricular length at diastole; HR, heart rate; LVCO, left ventricular cardiac output; BiP, biplane; LVIDs, left ventricular internal diameter at systole; ESV, end-systolic volume; f, frequency; P, power; AG, amplitude gain; Compr, compression; DDP, depth dependent gain; D, depth; FPS, frames per second; G, gain; PFR, pulse repetition frequency; SV, sample volume; Rej, reject; SVD, stress velocity diastolic; ACE, angle correction enabled; TR, tricuspid regurgitation, RV FAC, right ventricular fractional area change; RVA(s), right ventricular area at the end of systole; RVA(d), right ventricular area at the end of diastole; AVC, aortic valve closure; APLAX, apical long-axis view; ANT, anterior; SEPT, septal; INF, inferior; LAT, lateral; POST, posterior; GLPS, global longitudinal peak strain; LAX, long-axis; A4C, apical four-chamber view; A2C, apical two-chamber view; AVG, average; AVC_STORED, stored aortic valve closure; FR, frame rate; PSD, post-systolic duration; S_R, left atrial reservoir strain; S_CD, left atrial conduit strain; S_CT, left atrial contraction strain; LAV, left atrial volume; PreA, the time point before the mitral late filling-wave. “V” in the images in an image marking, the position of this marking corresponds to the marking on the transducer, helping physicians identify the orientation in the image.

This review offers a detailed overview of published evidences regarding the prognostic relevance of pre-operative echocardiographic parameters in patients undergoing MV surgery for PMR. Key prognostic indicators are a LVESD >37–40 mm, LVEF <60%, LA diameter >55 mm or LAVi >53–60 mL/m^2^, resting SPAP >45–50 mmHg, impaired RV function, increasing grades of FTR, LV GLS <17.9%–20.5% (absolute value), LA reservoir strain <21%–22%, exercise-induced parameters and finally 3D echocardiography. By utilizing these parameters, one can predict post-operative outcomes such as survival, cardiac events, LVEF, and cardiac reverse remodeling, with survival being of utmost importance. To aid clinical cardiologists in patient communication and risk estimation, echocardiographic indicators associated with short-, mid-, and long-term post-operative survival are summarized in Fig. 2 (Ref. [12, 15, 16, 37, 38, 39, 48, 51, 53, 54, 55, 57, 58, 59, 63, 64, 66, 67, 70, 72, 74, 79, 80, 83, 85, 89]), serving as a practical tool.

## 7. Conclusions

Echocardiography plays a critical role in patients with PMR, the prognostic value of echocardiographic parameters was comprehensively summarized and elucidated in this review, including the traditional parameters and novel parameters. This review may help facilitate the integration of these parameters into the clinical decision-making process and further improve the outcomes of patients with PMR. In the future, further larger, multi-centered, prospective studies and randomized clinical trials are still required to clarify and strengthen their precise values.

## Supplementary Material

Supplementary material associated with this article can be found, in the online version, at https://doi.org/10.31083/j.rcm2511414.
