# Supplementary material for: Prognostic Value of Pre-Operative Transthoracic Echocardiography in Patients with Primary Mitral Regurgitation
Source: Rev Cardiovasc Med. 2024 Nov 21;25(11):414. doi: 10.31083/j.rcm2511414 (PMC11607497; doi:10.31083/j.rcm2511414)
Supplement: Supplementary file 1 [file 2153-8174-25-11-414-s1.docx]

Supplementary Table 1. Description of included studies of left ventricular end-systolic diameter.

| First Author [Ref.]; Year Published; *Journal* | Total Number of Participants | Age | Female (%) | Population (severity/symptom) | Surgery | Reproducibility (interobserver/intraobserver) | Baseline LVEF (%) | Baseline LVESD (mm) | Outcomes | Follow-up | Association Between LVESD and Outcomes | Receiver-Operating Characteristic Curve Analysis |
| --- | --- | --- | --- | --- | --- | --- | --- | --- | --- | --- | --- | --- |
| Tribouilloy *et al.* [15], 2009, *J Am Coll Cardiol* | 739 | 64.8 ± 11.9 | 30 | Moderate to severe or severe/both | Surgery 74.7% (repair 78%, replacement 22%) + conservative (25.3%) | NA | 65.3 ± 10.3 | 35.8 ± 7.1 | All-cause mortality | Surgical population: 5.2 ± 3.4 years | For the subgroup after mitral valve surgery:  Larger LVESD as continuous variable on multivariable Cox survival analysis was associated with higher post-operative all-cause mortality (adjusted HR 1.03 [1.01–1.07] per 1 mm increment, *p* = 0.006); LVESD ≥40 mm (based on ACC/AHA guidelines recommendation) as categorical variable on multivariable Cox survival analysis was associated with more frequent post-operative all-cause mortality (adjusted HR 1.86 [1.11–3.15], *p* = 0.019). | NA |
|  |  |  |  |  |  |  |  |  | Cardiac mortality | Surgical population: 5.2 ± 3.4 years | For the subgroup after mitral valve surgery:  Larger LVESD as continuous variable on multivariable Cox survival analysis was associated with more frequent post-operative death from cardiac causes (adjusted HR 1.05 [1.01–1.10] per 1 mm increment, *p* = 0.01); LVESD ≥40 mm (based on ACC/AHA guidelines recommendation) as categorical variable on multivariable Cox survival analysis was associated with post-operative death from cardiac causes (adjusted HR 1.81 [1.05–3.54], *p* = 0.04). | NA |
| Song *et al.* [23], 2011, *Annals of Thoracis Surgery* | 147 | 48 ± 15 | 39 | Severe/both | Surgery (repair 100%) | Interobserver: ICC 0.962 (*p* < 0.001), CV 4.3%;  Intraobserver: NA | 63 ± 6.5 | 38.8 ± 6.0 | LVEF <55% | within 7 days | Larger LVESD on multivariate linear regression model was associated with poorer post-operative LVEF (β = –0.428 [–0.713 to –0.143], *p* = 0.004). | LVESD cut-off value 41 mm to predict severe LV dysfunction (LVEF <35%) with a sensitivity of 100% and a specificity of 69%. LVESDi cut-off value 24.9 mm/m^2^ to predict severe LV dysfunction (LVEF <35%) with a sensitivity of 100% and a specificity of 78%. |
|  |  |  |  |  |  |  |  |  | LVEF <35% | within 7 days | Receiver-operating characteristic curve analysis for predicting severe LV dysfunction showed that area under the curve was 0.899 (0.826–1.000). |  |
|  |  |  |  |  |  |  |  |  | Late follow-up LVEF | >3 months | Larger LVESD on multivariate linear regression model was an independent predictor of late post-operative LVEF (β = –0.248 [–0.413 to –0.049], *p* = 0.004). | NA |
|  |  |  |  |  |  |  |  |  | Cardiac events (death/hospital admission for heart failure) | 21 ± 17 months (1.8 ± 1.4 years) | Larger LVESD on multivariable Cox survival analysis was associated with higher clinical events (HR 1.26 [1.11–1.44] per 1 mm increment, *p* < 0.001). | NA |
| Tribouilloy *et al.* [22], 2011, *Eur J Echocardiogr* | 335 | 65 ± 11 | 30 | Severe/both | Surgery (repair 100%) | NA | 68 ± 9 | 37 ± 7 | LVEF <50% | 10.8 (9.1–12.0) months | Larger LVESD as continuous variable on multivariate logistic regression was an independent predictor of post-operative LV dysfunction (adjusted OR 1.03 [1.02–1.07] per 1 mm increment, *p* = 0.03).  LVESD ≥37 mm or/and LVEF <64% as categorical variable on multivariate logistic regression were associated with post-operative LV dysfunction (OR 2.63 [1.29–5.40], *p* = 0.008; OR 3,78 [1.88–7.63], *p* < 0.001).  Incremental prognostic value of LVESD ≥37 mm or/and LVEF <64% was demonstrated (AUC 0.64→0.74→0.83 [*p* < 0.001]). | LVESD cut-off value 37 mm to predict LV dysfunction (AUC 0.69 [0.58–0.76]). |
| Mascle *et al*. [21], 2012, *J Am Soc Echocardiogr* | 88 | 63 ± 13 | 33 | Severe/both | Surgery (repair 81.8%, replacement 18.2%) | Interobserver: absolute difference 0.7 ± 0.7;  Intraobserver: absolute difference 0.4 ± 0.5 | 66 ± 7 | LVESD: 36 ± 6.1 (mm);  LVESDi: 19.7 ± 3.6 (mm/m^2^); | LVEF <50% | 6 ± 1 months | Larger LVESD on univariate linear regression analysis was associated with worse post-operative LV function (r = –0.46, *p* < 0.001);  LVESDi ≥22 mm/m^2^ on multivariate logistic regression was associated with post-operative LV dysfunction (OR 4.2 [1.4–13], *p* = 0.02). | LVESDi cut-off value 22 mm/m^2^ to predict LV dysfunction with a sensitivity of 53% and a specificity of 78% (AUC 0.71). |
| Witkowski *et al*. [20], 2013, *Eur Heart J Cardiovasc Imaging* | 233 | 61 ± 12 | 39 | Moderate to severe/both | Surgery (repair 100%) | NA | 66 ± 9 | 31 ± 6 | LVEF <50% | 34 ± 20 months (2.8 ± 1.7 years) | Larger LVESD on multivariate linear regression analysis was associated with poorer post-operative LV function (*p* < 0.001 of all models);  LVESD ≥40 mm on multivariate logistic regression was associated with post-operative LV dysfunction (OR 6.71 [1.91–23.52], *p* = 0.003). | NA |
| Kitai *et al.* [19], 2014, *J Thorac Cardiovasc Surg* | 473 | 57 ± 14 | 45 | Severe/both | Surgery (repair 100%) | NA | 64 ± 10 | 35 ± 8 | LVEF <50% | 3 years | Larger LVESD on multivariate Cox survival analysis was independent predictor of post-operative LV dysfunction (OR 1.11 [1.05–1.17], *p* < 0.001). | LVESD cut-off value 39 mm or greater to predict LV dysfunction (AUC 0.724 [0.650–0.799], *p* < 0.001). |
| Candan *et al.* [18], 2017, *Echocardiography* | 59 | 46.7 ± 13 | 46 | Severe/asymptomatic | Surgery (repair 79%, replacement 21%) | NA | 65.2 ± 8 | 34.7 ± 8.1 | LVEF <50% | 6 months | Larger LVESD on univariate linear regression analysis was associated with poorer post-operative LVEF (r = –0.59; *p* < 0.001);  LVESD on multivariate logistic regression analysis was associated with post-operative LV dysfunction (adjusted OR 5.3 [1.6–17.8], *p* = 0.006). | NA |
| Zhou *et al.* [16], 2018, *Ann Thorac Surg* | 1903 | 54.2 ± 13.1 | 31 | Severe/both | Surgery (repair 100%) | NA | 65.8 ± 7.1 | 36.3 ± 6.4 | All-cause mortality | 3.8 ± 2.7 years | LVESD >40 mm on multivariate Cox survival analysis was associated with higher post-operative all-cause mortality (HR 6.78 [2.88–15.73], *p* < 0.001). | NA |
|  |  |  |  |  |  |  |  |  | Recurrent MR | 3.8 ± 2.7 years | LVESD >40mm on univariate Cox survival analysis was associated with more frequent recurrent MR (HR 2.83 [1.51–3.74], *p* < 0.001), but on multivariate Cox survival analysis, LVESD >40 mm was not associated with recurrent MR. | NA |
| Li *et al.* [14], 2020, *J Cardiothorac Surg* | 322 | 52.7 ± 12.7 | 22 | Severe/both and patients with LV dysfunction (LVEF <60% and LVESD >40 mm) | Surgery (repair 100%) | NA | 58.7 ± 7.3 | 43.9 ± 5.9 | Any decrease in LVEF | 3.1 ± 2.6 years | LVESD ≥45 mm on multivariate logistic regression was associated with post-operative deteriorated LV function (OR 2.95 [1.63–5.34], *p* < 0.001). | LVESD cut-off value 44.5 mm to predict deteriorated LV function (AUC 0.643 [0.563–0.724]). |

The Cell colors conform with Fig. 1. LVESD, left ventricular end-systolic diameter; LVESDi, left ventricular end-systolic diameter index; LV, left ventricle; LVEF, left ventricular ejection fraction; ICC, intraclass correlation coefficients; CV, coefficient of variation; NA, not available; AUC, area under curve; ACC/AHA, American College of Cardiology/American Heart Association; MR, mitral regurgitation; OR, odds ratio; HR, hazard ratio.

Supplementary Table 2. Description of included studies of left ventricular ejection fraction.

| First Author [Ref.]; Year Published; *Journal* | Total Number of Participants | Age | Female (%) | Population (severity/symptom) | Surgery | Reproducibility (interobserver/intraobserver) | Baseline LVEF (%) | Outcomes | Follow-up | Association Between LVEF and Outcomes | Receiver-Operating Characteristic Curve Analysis |
| --- | --- | --- | --- | --- | --- | --- | --- | --- | --- | --- | --- |
| Song *et al.* [23], 2011, *Annals of Thoracis Surgery* | 147 | 48 ± 15 | 39 | Severe/both | Surgery (repair 100%) | Interobserver: ICC 0.962 (*p* < 0.001), CV 4.3%;  Intraobserver: NA | 63 ± 6.5 | LVEF | >3 months | LVEF on multivariate linear regression model was associated with post-operative LV function (β = 0.191 [0.035–0.348], *p* = 0.017). | NA |
| Tribouilloy *et al.* [22], 2011, *Eur J Echocardiogr* | 335 | 65 ± 11 | 30 | Severe/both | Surgery (repair 100%) | NA | 68 ± 9 | LVEF <50% | 10.8 (9.1–12.0) months | Less LVEF as continuous variable on multivariate logistic regression was associated with post-operative LV dysfunction (adjusted OR 1.02 [1.01–1.04] per 1% decrement, *p* = 0.04).  LVEF <64% or/and LVESD ≥37 mm as categorical variable on multivariate logistic regression were associated with post-operative LV dysfunction (OR 2.63 [1.29–5.40], *p* = 0.008; OR 3,78 [1.88–7.63], *p* < 0.001).  Incremental prognostic value of LVEF <64% or/and LVESD ≥37 mm was demonstrated (AUC 0.64→0.74→0.83, *p* < 0.001). | LVEF cut-off value 64% or less to predict LV dysfunction (AUC 0.69 [0.58–0.76]). |
| Kitai *et al.* [19], 2014, *J Thorac Cardiovasc Surg* | 473 | 57 ± 14 | 45 | Severe/both | Surgery (repair 100%) | NA | 64 ± 10 | LVEF <50% | 3 years | Less LVEF on multivariate Cox survival analysis was associated with post-operative LV dysfunction (OR 0.93 [0.90–0.96], *p* < 0.001). | LVEF cut-off value 63% or less to predict LV dysfunction (AUC 0.725 [0.652–0.798], *p* < 0.001). |
| Machado *et al.* [27], 2014, *Cardiovasc Ultrasound* | 62 | 52.5 ± 1.3 | 40 | Severe/both | Surgery (repair 54.8%, replacement 45.2%) | Interobserver: ICC 0.97 (*p* < 0.0001), CV 8.7% (*p* = 0.10);  Intraobserver: ICC 0.98 (*p* < 0.0001), CV 9.3% (*p* = 0.10). | 66.1 ± 7.5 | LARR: LAVi reduction ≥25% | 2–10 months | Larger LVEF on multivariate logistic regression analysis was associated with LARR (OR 1.086 [1.002–1.178]). | LVEF cut-off value 63.5% to predict post-operative LARR with a sensitivity of 72% and specificity of 56%. |
| Zhou *et al.* [16], 2018, *Ann Thorac Surg* | 1903 | 54.2 ± 13.1 | 31 | Severe/both | Surgery (repair 100%) | NA | 65.8 ± 7.1 | All-cause mortality | 3.8 ± 2.7 years | LVEF <60% on univariate Cox survival analysis was associated with all-cause mortality (HR 2.43 [1.10–5.36], *p* = 0.03), but on multivariate Cox survival analysis, LVEF <60% was not associated with post-operative all-cause mortality. | NA |
|  |  |  |  |  |  |  |  | Recurrent MR | 3.8 ± 2.7 years | LVEF <60% on multivariate Cox survival analysis was independent predictor of recurrent MR (HR 4.01 [2.24–7.17], *p* < 0.001). | NA |
| Li *et al.* [14], 2020, *J Cardiothorac Surg* | 322 | 52.7 ± 12.7 | 22 | Severe/both, and patients with LV dysfunction | Surgery (repair 100%) | NA | 58.7 ± 7.3 | Any decrease in LVEF | 3.1 ± 2.6 years | LVEF ≤52% on multivariate logistic regression was associated with post-operative deteriorated LV dysfunction (OR 2.79 [1.06–7.33], *p* = 0.04). | LVEF cut-off value 52.5% to predict deteriorated LV function (AUC 0.578 [0.496–0.659]). |
| Nair *et al.* [12], 2021, *Indian J Thorac Cardiovasc Surg* | 244 | 48 ± 12.3 | 30 | Severe/both | Surgery (repair 100%) | NA | >60: 77.0%; 45–60: 12.7%: 35–44: 8.6%; <35: 1.6%. | In-hospital mortality | 7.1 (5–25) days | LVEF <60% on multivariate logistic regression was associated with in-hospital mortality (*p* = 0.04). | NA |
| Hu *et al.* [13], 2021, *Ann Thorac Surg* | 623 | 54.8 ± 12.1 | 41 | Severe/asymptomatic | Surgery (repair 100%) | NA | 67.2 ± 5.1 | LVEF <50% | 7–10 days | Less LVEF on multivariate logistic regression was associated with post-operative LV dysfunction (HR 0.933 [0.891–0.977], *p* = 0.003). | LVEF cut-off value 65% or less to predict LV dysfunction (AUC 0.765 [0.719–0.810]; *p* < 0.001) |

The Cell colors conform with Fig. 1. LV, left ventricle; LVEF, left ventricular ejection fraction; LAVi, left atrial volume index; LARR, left atria reverse remodeling; ICC, intraclass correlation coefficients; CV, coefficient of variation; NA, not available; AUC, area under curve; OR, odds ratio; HR, hazard ratio.

Supplementary Table 3. Description of included studies of left atrial size and function.

| First Author [Ref.]; Year Published; *Journal* | Total Number of Participants | Age | Female (%) | Population (severity/symptom) | Surgery | Reproducibility (interobserver/sintraobserver) | Baseline LVEF (%) | Baseline LA size | Outcomes | Follow-up | Association Between LA Size and Outcomes | Receiver-Operating Characteristic Curve Analysis |
| --- | --- | --- | --- | --- | --- | --- | --- | --- | --- | --- | --- | --- |
| Antonini-Canterin *et al.* [41], 2008, *Heart* | 79 | 60 ± 12 | 30 | Severe/symptomatic | Surgery (repair 100%) | Interobserver: absolute difference 3.1 ± 0.8%;  Intraobserver: NA | 62 ± 6 | LAVi: 68 ± 22 (mL/m^2^) | LARR: LAVi reduction | 1–6 months | Larger LAVi on multivariate linear regression analysis was independently associated with more significant post-operative LAVi reduction (*p* < 0.001). | NA |
| Song *et al.* [44], 2010, *Clin Cardiol* | 85 | 54.7 ± 13.3 | 62 | Severe/both | Surgery (repair 100%) | NA | 58.5 ± 9.3 | LAVi: 75.3 ± 33.5 (mL/m^2^) | LARR: LAVi reduction | Immediately;  6 and 12 months after surgery. | Larger LAVi on multivariate linear regression analysis was independently associated with less significant post-operative LAVi reduction (β = –0.595, *p* = 0.001). | NA |
| Candan *et al.* [42], 2014, *Int J Cardiovasc Imaging* | 53 | 45.7 ± 13.5 | 55 | Severe/both | Surgery (repair 56.6%, replacement 43.4%) | Interobserver: ICC –2.7% (–1.9%–7.3%);  Intraobserver: ICC 0.90% (0.84%–0.83); | 64.6 ± 7.1 | LAVi: 58.2 ± 15.7 (mL/m^2^) | LARR: LAVi reduction | >6 months | Larger LAVi on multivasriate linear regression analysis was associated with more significant post-operative LAVi reduction (β = 0.64, *p* < 0.001). | NA |
| Di Gioia *et al.* [40], 2017, *Int J Cardiol* | 134 | 59.6 ± 3.3 | 27 | Severe/both | Surgery (repair 87.3%, replacement 12.7%) | NA | 60 ± 1 | LAVi: 50.7 ± 3.5 (mL/m^2^) | Early post-operative atrial fibrillation, late atrial fibrillation | within 2 weeks and more than 2 weeks after surgery | Larger LAVi on multivariate logistic regression analysis was associated with more frequent early atrial fibrillation (OR 1.03 [1.00–1.06]; *p* = 0.01) and late post-operative atrial fibrillation (HR 1.03 [1.00–1.06], *p* = 0.02). | NA |
|  |  |  |  |  |  |  |  |  | LARR and LV reverse remodeling: LAVi reduction >15% and any reduction of LV mass | 42 ± 16 months (3.5 ± 1.3 years) | Larger LAVi on multivariate logistic regression analysis was associated with more frequent atrial and ventricular reverse remodeling (OR 1.04 [1.01–1.07], *p* = 0.01). | NA |
| Kim *et al.* [39], 2018, *JACC Cardiovasc Imaging* | 506 | 58.5 ± 13.7 | 46 | Severe/both | Surgery (repair 75%, replacement 25%) | Interobserver: ICC 0.96;  Intraobserver: NA | 59.9 ± 8.2 | LA dimension: 56.4 ± 2.0 (mm) | Cardiac events (cardiac death, worsening heart failure, reoperation for failure of MV surgery) | 3.5 (1.3–6.3) years | Larger LA dimension on multivariate Cox analysis was associated with more frequent cardiac events (HR 1.034 [1.006–1.063], *p* = 0.019). | NA |
|  |  |  |  |  |  |  |  |  | All-cause mortality | 3.5 (1.3–6.3) years | Larger LA dimension on multivariate Cox analysis was associated with all-cause mortality (HR 1.031 [1.005–1.058], *p* = 0.019). | NA |
| Essayagh *et al.* [38], 2019, *J Am Coll Cardiol* | 5769 (1405 patients underwent mitral valve surgery) | 63 ± 16 | 47 | All patients with a diagnosis of degenerative mitral disease | surgery 24% (repair 92%, replacement 8%) + conservative (76%) | NA | 63 ± 8 | Overall LAVi: 43 ± 24 (mL/m^2^) | Post-operative survival (secondary endpoints) | 6.8 ± 3.1 years | For the subgroup after mitral surgery: Higher LAVi on univariate Cox survival analysis was associated with higher mortality (As continuous variable: HR 1.08 [1.04–1.13], *p* = 0.0002 per 10 mL/m^2^; As categorical variable defined in previous studies of mitral diseases: HR 1.65 [1.10–2.55], *p* = 0.02 for LAVi ≥60 mL/m^2^ vs. <40 mL/m^2^ and HR 1.75 [1.23–2.52], *p* < 0.0017 vs. LAVi 40 to 59 mL/m^2^).  Higher LAVi on multivariate Cox survival analysis as continuous variable was associated with higher mortality (HR 1.05 [1.00–1.11], *p* = 0.05 per 10 mL/m^2^); as categorical variable was not associated with mortality (the link between LAVi and mortality was significant [log-rank *p* < 0.0026] but not large and was mostly for severe LA enlargement [10-year survival 85 ± 3% vs. 86 ± 2% and 75 ± 3% for LAVI <40, 40 to 59, and ≥60 mL/m^2^, respectively; *p* = 0.0026]).  Incremental prognostic value of LAVi was demonstrated (*p* = 0.05 per 10 mL/m^2^). | NA |
| Szymanski *et al.* [37], 2019, *Cardiology* | 305 | 62.6 ± 0.2 | 37 | severe/both | Surgery (repair 100%) | Interobserver: ICC 0.94;  Intraobserver: ICC 0.96 | 67.9 ± 0.8 | LA area: 29.4 ± 5.4 (cm^2^) | All-cause mortality | 8.2 ± 3.4 years | Larger LA area (>30 cm^2^) on multivariable Cox survival analysis was associated with higher mortality compared to LA area ≤25 cm^2^ (adjusted HR 2.20 [1.03–4.90], *p* = 0.042). | NA |
| Balachandran *et al.* [43], 2020, *J Thorac Cardiovasc Surg* | 720 | 61.1 (52.7–69.1) | 28 | severe/both | Surgery (repair 100%) | NA | 62 (58–65) | LAVi: 54.0 (44.0–66.0) (mL/m^2^) | LARR: LAVi reduction | 4.5 (0.1–21.8) months | Larger LAVi on multivariable repeated-measures analysis was associated with greater LARR despite their LAVi level remaining the highest (worst) post-operatively (*p* < 0.001). | NA |
|  |  |  |  |  |  |  |  |  | Atrial fibrillation | <1 months | Larger LAVi on a flexibly fitted logistic regression model was associated with more frequent early post-operative atrial fibrillation (*p* = 0.030). | NA |
|  |  |  |  |  |  |  |  |  | Mortality | 4.6 (3.3–5.9) years | Baseline LAVi on Cox survival analysis was not associated with long-term mortality statistically (adjusted HR 2.35 [0.88–6.27], *p* = 0.077), but a potential association was suggested. LAVi >50 mL/m^2^ and <40 mL/m^2^ on graphical exploration were associated with higher risk of 5-year mortality. | NA |
| Hu *et al.* [13], 2021, *Ann Thorac Surg* | 623 | 54.8 ± 12.1 | 41 | severe/asymptomatic | Surgery (repair 100%) | NA | 67.2 ± 5.1 | LAVi: 51.2 ± 3.5 (mL/m^2^) | LVEF <50% | 7 to 10 days | Larger LAVi on multivariate logistic regression was associated with post-operative LV dysfunction (HR 1.212 [1.106–1.329], *p* < 0.001). | LAVi cut-off value 53 mL/m^2^ to predict LV dysfunction (AUC 0.733 [0.683–0.784]; *p* < 0.001). |
| Essayagh *et al.* [48], 2022, *J Am Heart Assoc* | 4792 (1106 patients underwent mitral valve surgery) | 61 ± 16 | 48 | both/both | surgery 24% (repair 94%, replacement 6%) + conservative (76%) | NA | 63 ± 7 | LA coupling index: 5.8 ± 3.7 (LAVi/tissue Doppler imaging a′) | Post-operative survival (secondary endpoints) | 7 ± 3 years | For the subgroup after mitral surgery: Higher LA coupling index on univariable Cox survival analysis was associated with higher mortality (As continuous variable: HR 1.23 [1.13–1.31], *p* < 0.0001 per 3 units; As categorical variable defined in Spline curve analysis: HR 2.17 [1.27–3.71], *p* = 0.005 vs LA coupling index <5);  Higher LA coupling index on multivariate Cox survival analysis as continuous variable was associated with higher mortality (HR 1.13 [1.00–1.24], *p* = 0.04 per 3 units); as categorical variable was not significantly associated with mortality.  The incremental prognostic value of LA coupling index was demonstrated by several approaches. | NA |

The Cell colors conform with Fig. 1. LA, left atrium; LV, left ventricle; LVEF, left ventricular ejection fraction; LAVi, left atrial volume index; LARR, left atria reverse remodeling; ICC, intraclass correlation coefficients; CV, coefficient of variation; NA, not available; AUC, area under curve; LV GLS, left ventricular global longitudinal strain; OR, odds ratio; HR, hazard ratio.

Supplementary Table 4. Description of included studies of pulmonary hypertension.

| First Author [Ref.]; Year Published; *Journal* | Total Number of Participants | Age | Female (%) | Population (severity/symptom) | Surgery | Reproducibility (interobserver/intraobserver) | Baseline LVEF (%) | Baseline SPAP (mmHg) | Outcomes | Follow-up | Association Between Pulmonary Hypertension and Outcomes | Receiver-Operating Characteristic Curve Analysis |
| --- | --- | --- | --- | --- | --- | --- | --- | --- | --- | --- | --- | --- |
| Yang *et al.* [61], 2006, *J Am Soc Echocardiogr* | 79 | 64 ± 14 | 35 | moderate to severe or severe/both | Surgery (repair 68%, replacement 32%) | NA | 61 ± 11 | Catheterization-derived: 49 ± 14 | LVEF <50% | 31 ± 17 days | Higher SPAP on multivariate linear regression model was associated with poorer post-operative LVEF. (β = –0.53, *p* = 0.0001). | NA |
| Le Tourneau *et al.* [54], 2010, *Heart* | 256 | 63 ± 12 | 34 | moderate to severe or severe/both | Surgery (repair 76%, replacement 24%) | Interobserver: absolute difference 3.6 ± 1.9%;  intraobserver: absolute difference 1.8 ± 1.0%. | 65 ± 10 | Echocardiography-derived: 45 ± 14 | All-cause mortality | 4.1 ± 3.4 years | Higher SPAP as continuous variable on multivariable Cox survival analysis was independent predictor of higher all-cause mortality (HR 1.43 [1.09–1.88] per 10 mmHg increment, *p* = 0.011).  SPAP ≥50 mmHg on multivariable Cox survival analysis was associated with higher all-cause mortality (HR 2.58 [1.21–5.45], *p* = 0.014).  Incremental prognostic value of SPAP was demonstrated (χ^2^ 13→35, *p* = 0.011). | SPAP cut-off value 50 mmHg to predict all-cause mortality with a sensitivity of 61% and a specificity of 72% (AUC 0.7, *p* < 0.0001); and cut-off value 45 mmHg to predict all-cause mortality with a sensitivity of 71% and a specificity of 62% (AUC 0.7, *p* < 0.0001). |
|  |  |  |  |  |  |  |  |  | Cardiovascular mortality | 4.1 ± 3.4 years | Higher SPAP as continuous variable on multivariable Cox survival analysis was associated with higher cardiovascular mortality (HR 1.49 [1.03–2.16] per 10 mmHg increment, *p* = 0.033).  SPAP ≥50 mmHg on multivariable Cox survival analysis was associated with cardiovascular mortality (HR 2.47 [1.01–6.88], *p* = 0.05). | NA |
| Ghoreishi *et al.* [55], 2011, *J Thorac Cardiovasc Surg* | 873 | 59 ± 14 | 41 | ≥ moderate/both | Surgery (repair 86%, replacement 14%) | NA | 52 ± 14 | Echocardiography-derived: 46 ± 15  Right heart catheterization-derived: 43 ± 17 | Operative mortality (in-hospital mortality or death within 30 days of operation) | 1 months | Higher SPAP as continuous variable on multivariate logistic regression was associated with higher operative mortality (OR 1.023 [1.003–1.044] per 1 mmHg increment, *p* = 0.027). | NA |
|  |  |  |  |  |  |  |  |  | All-cause mortality | 35 ± 24 months  (2.9 ± 2 years) | Higher SPAP as continuous variable on multivariable Cox survival analysis was associated with higher late mortality (HR 1.018 [1.007–1.028] per 1 mmHg increment, *p* = 0.001). | NA |
| Barbieri *et al.* [53], 2011, *Eur Heart J* | 437 | 67.5 ± 11.4 | 34 | moderate to severe/both | surgery 74.4% (repair 78%, replacement 22%) + conservative (25.6%) | NA | 64.1 ± 10.1 | Echocardiology-derived: 45 ± 15 | All-cause mortality | 4.8 ± 2.8 years | SPAP >50 mmHg on multivariate Cox survival analysis was associated with higher post-operative mortality (HR 2.15 [1.20–3.83], *p* = 0.01). | NA |
|  |  |  |  |  |  |  |  |  | Cardiovascular mortality | 4.8 ± 2.8 years | SPAP >50 mmHg on multivariate Cox survival analysis was associated with higher cardiovascular death (HR 2.46 [1.24–4.88], *p* = 0.01). | NA |
| Nozohoor *et al.* [59], 2012, *J Card Surg* | 270 | 62.9 ± 2.2 | 34 | severe/both | Surgery (repair 75.2%, replacement 24.8%) | NA | ≥50%: 87%  30–49%: 11.9%  <30%: 1.1% | Echocardiology-derived: PH (+, SPAP >50 mmHg): 74 (27.4%)  PH (-): 196 (72.6%) | 30-day mortality | 1 month | PH (SPAP >50 mmHg, based on current guidelines) was not associated with 30-day mortality (log-rank *p* = 0.550). | NA |
|  |  |  |  |  |  |  |  |  | All-cause mortality | <3 years | PH on multivariate Cox survival analysis was associated with higher mortality (HR 4.3 [1.08–17.4], *p* = 0.01). | NA |
|  |  |  |  |  |  |  |  |  | All-cause mortality | 3–5 years | PH on multivariate Cox survival analysis was not associated with mortality (HR 0.13 [0.02–1.1], *p* = 0.056). | NA |
|  |  |  |  |  |  |  |  |  | All-cause mortality | >5 years | PH on multivariate Cox survival analysis was associated with higher mortality (HR 0.13 [0.03–0.60], *p* = 0.008). |  |
| Varghese *et al.* [60], 2014, *J Thorac Cardiovasc Surg* | 632 | 57 ± 13 | 36 | severe/both | Surgery (repair 100%) | NA | 60.7 ± 7.5 | Catheterization or echocardiography-derived: 34.6 ± 13.3 | LVEF <50% | 4.1 days | Higher SPAP as continuous variable on multivariate linear regression was associated with post-operative LV dysfunction (β = –0.167, *p* < 0.001).  SPAP >50 mmHg on multivariate logistic regression was associated with post-operative LV dysfunction (OR 1.95 [1.22–3.10], *p* = 0.005). | NA |
| Coutinho *et al.* [51], 2015, *Eur J Cardiothorac Surg* | 382 | 55.7 ± 4.7 | 28 | severe/asymptomatic | Surgery (repair 98.2%, replacement 1.8%) | NA | 68.3 ± 0.5 | catheterization or echocardiography-derived: 42.7 ± 9.0 | All-cause mortality | 6.9 (3.8–11.5) years | Atrial fibrillation/SPAP >50 mmHg on multivariate Cox survival analysis was associated with higher late mortality (HR 2.32 [1.13–4.78], *p* = 0.02). | NA |
|  |  |  |  |  |  |  |  |  | Reoperation | 6.9 (3.8–11.5) years | Atrial fibrillation/SPAP >50 mmHg on multivariate Cox survival analysis was associated with higher risk of reoperation (HR 4.20 [1.10–11.20], *p* = 0.03). | NA |
| Murashita *et al.* [58], 2015, *Ann Thorac Cardiovasc Surg* | 654 | 56.4 ± 2.8 | 41 | NA/both | Surgery (repair 100%) | NA | 66.6 ± 0.9 | Echocardiography-derived:  PH (+, SPAP >50 mmHg): 137  PH (-): 571 | 30-day mortality | 1 months | PH (SPAP >50 mmHg, based on current guidelines) was not associated with 30-day mortality (log-rank *p* = 0.975). | NA |
|  |  |  |  |  |  |  |  |  | Late all-cause mortality | 7.5 years | PH was associated with higher late all-cause mortality (log-rank *p* = 0.019). | NA |
|  |  |  |  |  |  |  |  |  | Major adverse cardiac and cerebrovascular events | 7.5 years | PH was not associated with major adverse cardiac and cerebrovascular events (log-rank *p* = 0.262). | NA |
| Mentias. *et al.* [57], 2016, *J Am Coll Cardiol* | 1318 | 62 ± 13 | 34 | moderate to severe or severe/both | surgery 86% (repair 92%, replacement 8%) + conservative (14%) | NA | 63 ± 2 | Echocardiography-derived: 37 ± 14 | All-cause mortality | 7.1 ± 2.0 years | SPAP as continuous variable on multivariable Cox survival analysis was associated with higher all-cause mortality (HR 1.23 [1.12–1.36] per 10 mmHg increment, *p* < 0.01).  The incremental prognostic value of SPAP for mortality was demonstrated (χ^2^ 74→153, *p* < 0.001). | NA |
|  |  |  |  |  |  |  |  |  | Post-operative SPAP (≥35 mmHg) | pre-discharge | SPAP as continuous variable on multivariate linear regression was associated with post-operative SPAP (β = 0.28, *p* < 0.001).  Higher SPAP on multivariate logistic regression was associated with elevated post-operative SPAP (Wald statistic 24.7, *p* < 0.001). | NA |
| Genuardi *et al.* [56], 2021, *J Am Heart Assoc* | 488 | 67.2 ± 11.2 | 45 | severe/asymptomatic | Surgery (repair 69.5%, replacement 30.5%) | NA | 49.0 ± 1.9 | Catheterization-derived: mean pulmonary artery pressure 31.9 ± 6.6 | All-cause mortality | 3.9 years | Higher mean pulmonary artery pressure as continuous variable on multivariable Cox survival analysis was associated with higher cardiovascular mortality (HR 1.38 [1.13–1.68] per 10 mmHg increment, *p* = 0.001). | NA |

The Cell colors conform with Fig. 1. SPAP, systolic pulmonary artery pressure; PH, pulmonary hypertension; LVEF, left ventricular ejection fraction; ICC, intraclass correlation coefficients; CV, coefficient of variation; NA, not available; AUC, area under curve; OR, odds ratio; HR, hazard ratio.

Supplementary Table 5. Description of included studies of right ventricular size and function.

| First Author [Ref.]; Year Published; *Journal* | Total Number of Participants | Age | Female (%) | Population (severity/symptom) | Surgery | Reproducibility (interobserver/intraobserver) | Baseline LVEF (%) | Baseline RV size and function | Outcomes | Follow-up | Association Between RV size or function and Outcomes | Receiver-Operating Characteristic Curve Analysis |
| --- | --- | --- | --- | --- | --- | --- | --- | --- | --- | --- | --- | --- |
| Haddad *et al.* [63], 2007, *J Am Soc Echocardiogr* | 50 | 67 ± 9 | 46 | Aortic or Mitral stenosis or regurgitation | Surgery (Mitral valve replacement 24%, Mitral valve repair 20%) | RV FAC:  Interobserver: absolute difference 4.2 ± 2.6%;  Intraobserver: absolute difference 2.9 ± 2.0%, ICC 0.95  RV MPI:  Interobserver: absolute difference 0.04 ± 0.02;  Intraobserver: absolute difference: 0.03 ± 0.02, ICC 0.97 | 56 ± 12 | RV FAC: 42 ± 12%;  RV MPI: 0.45 ± 0.17 | In-hospital mortality or circulatory failure | NA | Small RV FAC as continuous variable on multivariate logistic regression was associated with higher in-hospital mortality or circulatory failure (OR 0.001 [<0.001–0.727] per 1% increment, *p* = 0.048);  RV MPI ≥0.50 as categorical variable on multivariate logistic regression was associated with in-hospital mortality or circulatory failure (OR 25.20 [5.24–121.15], *p* < 0.0001) | RV MPI cut-off value 0.50 to predict in-hospital mortality or circulatory failure with a sensitivity of 82.3% and a specificity of 84.4% (AUC 0.879 [0.781–0.977]). |
| Chrustowicz *et al.* [65], 2010, *Echocardiography* | 45 | 58 ± 10 | 47 | severe/symptomatic or with LV dysfunction | Surgery (replacement 100%) | NA | 55.3 ± 11 | TAPSE: 19.4 ± 4.3 mm  S': 10.3 ± 3 cm/s | Decrease in LVEF >10% | 6 months | Larger TAPSE on multivariate logistic regression was associated with a more frequent decrease in LVEF >10% after surgery (OR 1.5 [1.1–2.1], *p* = 0.01).  Larger S' on multivariate logistic regression was associated with more frequent LVEF reduction >10% after surgery (OR 1.8 [1.1–2.9], *p* = 0.01).  When both TAPSE and S' were entered in the multivariate analysis, TAPSE (OR 1.7, *p* = 0.01) and gender (OR 0.04, *p* = 0.01) were significant. | TAPSE cut-off value 17.5 mm to predict LVEF reduction >10% with a sensitivity of 90% and a specificity of 63% (AUC 0.77);  S' cut-off value 8.75 mm/s to predict LVEF reduction >10% with a sensitivity of 90% and a specificity of 63% (AUC 0.79). |
| Gackowski *et al.* [28], 2010, *Cardiol J* | 40 | 61 ± 9 | 50 | severe/both | Surgery (replacement 100%) | NA | 55.3 ± 11 | RVEDD: 28.7 ± 4 mm | Post-operative prolonged ICU stay (>3 days) because of the need for inotropic support | Until discharge | Larger RVEDD on multivariate linear regression analysis was associated with prolonged ICU stay (β = 0.31, *p* = 0.02). | RVEDD cut-off value 35 mm to predict prolonged ICU stay with a sensitivity of 81% and a specificity of 91% (AUC 0.64 [0.47–0.82]). |
| Le Tourneau *et al.* [64], 2013, *Circulation* | 208 | 62 ± 12 | 34 | severe/both | Surgery (repair 71.1%, replacement 28.9%) | NA | 62.5 ± 9.8 | RV EF: 40.4 ± 10.2 | Cardiovascular mortality | 7.1 ± 4.3 years | RV EF ≤35% (derived from previous literature) on multivariable Cox survival analysis was associated with higher cardiovascular mortality (HR 2.3 [1.1–5.3], *p* = 0.042). | NA |
| Ye *et al.* [66], 2014, *J Thorac Cardiovasc Surg* | 781 | 61.5 ± 5.9 | 41 | severe/both | Surgery (repair 90.7%, replacement 9.3%) | Interobserver: ICC 0.80;  intraobserver: ICC 0.87 | 58.3 ± 0.8 | RV MPI: NA | All-cause mortality | 4.4 ± 3 years | Higher RV MPI on multiphase hazard model was associated with late death (Coefficient ± SE: 1.2 ± 0.37, *p* = 0.001). | NA |
| Towheed *et al.* [67], 2021, *J Am Heart Assoc* | 269 | 67.4 ± 14.9 | 40 | NA | Surgery (121 underwent mitral valve surgery and 148 underwent aortic surgery) | Interobserver: ICCs of RV FAC, TAPSE, S', and RV MPI were 0.84, 0.92, 0.90, and 0.83, Variabilities were 4.85 ± 10.3%, 0.01 ± 0.24, 0.1 ± 2.29 and –0.01 ± 0.1;  Intraobserver: ICCs of RV FAC, TAPSE, S', and RV MPI were 0.89, 0.95, 0.99, and 0.98, Variabilities were 1.7 ± 9.2%， 0.07 ± 0.2, 0.05 ± 0.22 and 0.01 ± 0.04 | 53.7 ± 16.4 | RV FAC: 42 ± 12%;  TAPSE: 1.9 ± 0.6 cm;  S': 12.7 ± 4 cm/s;  RV MPI: 0.65 ± 1.9;  RV dP/dt: 545 ± 267 mmHg/s. | All-cause mortality | <1 month | RV dysfunction (at least 3 abnormal RV parameters out of the 5 (>50%) parameters including RV FAC <35%, TAPSE <16%, S' <10 cm/s, RV MPI >0.40 by pulsed Doppler and >0.55 by tissue Doppler, and RV dP/dt <400 mmHg/s) on multivariate logistic regression was associated with 30-day mortality (OR 3.5 [1.1–11.1], *p* = 0.03). | NA |
|  |  |  |  |  |  |  |  |  | Post-operative adverse events | <1 month | RV dysfunction was associated with 30-day composite adverse events (OR 4.2 [2.1–8.3], *p* < 0.01). | NA |

The Cell colors conform with Fig. 1. RV, right ventricle; LV, left ventricle; LVEF, left ventricular ejection fraction; RV FAC, right ventricular fractional area change; RV MPI, right ventricular myocardial performance index; TAPSE, tricuspid annular plane systolic excursion; RVEDD, right ventricular end-diastolic diameter; RV EF, right ventricular ejection fraction; RV FAC, right ventricular fractional area change; ICC, intraclass correlation coefficients; CV, coefficient of variation; NA, not available; AUC, area under curve; ACC/AHA, American College of Cardiology/American Heart Association; MR, mitral regurgitation; OR, odds ratio; HR, hazard ratio.

Supplementary Table 6. Description of included studies of functional tricuspid regurgitation.

| First Author [Ref.]; Year Published; *Journal* | Total Number of Participants | Age | Female (%) | Population (severity/symptom) | Surgery | Baseline LVEF (%) | Baseline FTR grades | Primary Outcome and Composite Cardiac Endpoint | Follow-up | Association Between FTR and Outcomes |
| --- | --- | --- | --- | --- | --- | --- | --- | --- | --- | --- |
| Chan *et al.* [70], 2009, *Ann Thorac Surg* | 624 | 63.5 ± 1.0 | 59 | ≥ moderate/both | Surgery (replacement 100%) | ≥50%: 61.9%  35–50%: 14.6%  20–34%: 7.4%;  <20%: 2.6% | Moderate to severe or severe: 37.0% | All-cause mortality | 6.8 ± 4.8 years | Moderate to severe or severe FTR on multivariable Cox survival analysis was associated with higher mortality (HR 1.5 [1.1–2.0], *p* = 0.003). |
|  |  |  |  |  |  |  |  | Congestive heart failure | 6.8 ± 4.8 years | Moderate to severe or severe FTR on multivariable Cox survival analysis was associated with congestive heart failure development (HR 2.2 [1.1–4.9], *p* = 0.04). |
| Murashita *et al.* [69], 2013, *Circ J* | 1138 | 56.4 ± 3.7 | 31 | NA/both | Surgery (repair 83.4%, replacement 16.6%) | 66.5 ± 0.3 | Mild or less: 73.2%;  moderate: 19.1%;  severe: 7.7%. | All-cause mortality | 7.5 ± 4.9 years | Severe pre-operative FTR was associated with poor survival compared with mild or less TR group or moderate TR group (log-rank *p* < 0.001). |
|  |  |  |  |  |  |  |  | Re-admission for congestive heart failure | 7.5 ± 4.9 years | Severe or moderate pre-operative FTR was associated with higher incidences of re-admission for congestive heart failure compared with mild or less TR group (log-rank *p* = 0.008). |
|  |  |  |  |  |  |  |  | Progression to severe tricuspid regurgitation | 7.5 ± 4.9 years | Pre-operative FTR on univariable Cox survival analysis was associated with recurrence of severe TR in patients without concomitant TR repair (HR 5.16 [1.78–14.9], *p* = 0.003). |
| Yeates A *et al.* [74], 2014, *ANZ J Surg* | 161 | 64 ± 12.6 | 55 | NA/both | Surgery (repair 37.3%, replacement 62.7%) | >55%: 76.4% | Mild or less: 65.2%;  moderate or severe: 34.8%. | All-cause mortality | 3.8 ± 2.7 years | Increasing grades of pre-operative FTR were associated with poor survival (adjusted log-rank *p* = 0.046). |
| David *et al.* [79], 2015, *J Thorac Cardiovasc Surg* | 1171 | 58.2 ± 18.7 | 30 | ≥ moderate/both | Surgery (repair 100%) | ≥60%: 65.5%  40–59%: 29.8%  20–39%: 4.3%;  <20%: 0.1% | Trivial: 43.8%;  mild: 44.4%;  moderate: 8.4%;  severe: 3.3%. | All-cause mortality | 9.1 ± 5.3 years | Moderate or severe FTR on multivariable Cox survival analysis was associated with higher post-operative mortality (pre-operative FTR with tricuspid annuloplasty vs no pre-operative FTR 1.93 [1.26–2.98], *p* = 0.003, pre-operative FTR without tricuspid annuloplasty vs no pre-operative FTR 1.92 [1.01–3.66], *p* = 0.05), pre-operative FTR without tricuspid annuloplasty vs pre-operative FTR with tricuspid annuloplasty 1.07 [0.50–2.25], *p* = 0.87). |
| Essayagh *et al.* [72], 2020, *Eur Heart J* | 5083 | 63 ± 16 | 47 | Both/both | surgery 24.5% (repair 90.1%, replacement 9.9%) + conservative (75.5%) | 63 ± 7 | Trivial: 45%;  mild: 37%;  moderate: 15%;  severe: 3%. | All-cause mortality | 6.8 ± 3.1 years | For the subgroup after mitral surgery:  FTR on multivariable Cox survival analysis was associated with post-operative survival (HR adjusted for left-sided variables: severe vs trivial 2.16 [1.04–4.50], *p* = 0.04; HR adjusted for right-sided variables: moderate vs trivial 2.11 [1.28–3.50], *p* = 0.004, severe vs trivial 2.90 [1.35–6.21], *p* = 0.006).  In patients underwent concomitant tricuspid surgery, pre-operative FTR don't have any association with post-operative survival (*p* = 0.76), while in those without tricuspid correction, pre-operative FTR severity has strongly association with post-operative survival (*p* < 0.0001). |

The Cell colors conform with Fig. 1. FTR, functional tricuspid regurgitation; LVEF, left ventricular ejection fraction; ICC, intraclass correlation coefficients; CV, coefficient of variation; NA, not available; AUC, area under curve; OR, odds ratio; HR, hazard ratio.

Supplementary Table 7. Description of included studies of left ventricular global longitudinal strain.

| First Author [Ref.]; Year Published; *Journal* | Total Number of Participants | Age | Female (%) | Population (severity/symptom) | Surgery | Vendor for echocardiography | Software for strain analysis | Reproducibility (interobserver/intraobserver) | Baseline LVEF (%) | Baseline LV GLS (%) | Primary Outcome and Composite Cardiac Endpoint | Follow-up | Association Between LV GLS and Outcomes | Receiver-Operating Characteristic Curve Analysis |
| --- | --- | --- | --- | --- | --- | --- | --- | --- | --- | --- | --- | --- | --- | --- |
| Song *et al.* [23], 2011, *Annals of Thoracis Surgery* | 147 | 48 ± 15 | 39 | severe/both | Surgery (repair 100%) | GE | EchoPAC | Interobserver: ICC 0.962 (*p* < 0.001), CV 4.3%.  Interobserver: NA | 63 ± 6.5 | –19.9 ± 4.0 | Cardiac events (Death/hospital admission for heart failure) or post-operative LVEF <55%) | Immediately/3 m/21 ± 17 m | Baseline LV GLS was not associated with post-operative LV dysfunction or clinical events. | NA |
| Donal *et al.* [86], 2012, *Eur Heart J Cardiovasc Imaging* | 77 | 63 ± 16 | 33 | severe/both | surgery (repair 93.5%) + conservative (6.5%) | GE | EchoPAC | Interobserver: CV 13.5% | 67 ± 12 | –18 ± 5 | LVEF <50% | 6 months | Worse LV GLS on univariate linear regression analysis was associated with poorer post-operative LV function (r = –0.422; *p* = 0.011);  Worse LV GLS on multivariate logistic regression analysis was associated with LV dysfunction (*p* = 0.028).  However, on the multivariate linear regression model, only exercise LV GLS normalized was associated with LV function (*p* = 0.019). | LV GLS cut-off value –18% to predict post-operative LV dysfunction with a sensitivity of 77% and specificity of 56% (AUC 0.685). |
| Florescu *et al.* [30], 2012, *Echocardiography* | 28 | 59 ± 13 | 36 | severe/asymptomatic | Surgery (repair 100%) | GE | EchoPAC | Interobserver: CV 6.1%;  Intraobserver: CV 1.8–2.5% | 63 ± 5 | –19.9 ± 4.4 | Decrease in LVEF >10% | 0.5 month | Worse LV GLS on univariate linear regression analysis was associated with poorer LV function; However, on multivariate linear regression model, only mean peak longitudinal systolic velocity was an independent predictor of a post-operative reduction of EF greater than 10% (r^2^ = 0.52, *p* < 0.001), and the combination of mean peak longitudinal systolic velocity and longitudinal systolic strain was the best predictor of a post-operative reduction of EF reduction greater than 10% (r^2^ = 0.70, *p* < 0.001). | LV GLS cut-off value –18% to predict a post-operative decrease in LVEF >10% with a sensitivity of 83% and specificity of 77% (AUC 0.96). |
| Mascle *et al.* [21], 2012, *J* *Am Soc Echocardiogr* | 88 | 63 ± 13 | 33 | severe/both | Surgery (repair 82%, replacement 18%) | GE | EchoPAC | Interobserver: absolute difference 1.4 ± 1.5%, relative difference 0.04%; Intraobserver: absolute difference 1.4 ± 1.1%, relative difference 0.05% | 66 ± 7 | –19.1 ± 3.6 | LVEF <50% | 6 months | Worse LV GLS on univariate linear regression analysis was associated with post-operative LV function (r = –0.29; *p* = 0.007).  Worse LV GLS than –18% on multivariate logistic regression was associated with post-operative LVEF <50% (adjusted OR 4.2 [1.4–13], *p* = 0.009). | NA |
| Witkowski *et al.* [20], 2013, *Eur Heart J Cardiovasc Imaging* | 233 | 61 ± 12 | 39 | moderate to severe or severe/both | Surgery (repair 100%) | GE | EchoPAC | Interobserver: absolute difference 0.9 ± 1.0;  Intraobserver: absolute difference 1.2 ± 0.5 | 66 ± 9 | –21.8 ± 4.1 | LVEF <50% | 34 ± 20 months (2.8 ± 1.7 years) | Worse LV GLS on multivariate logistic regression was associated with post-operative LV dysfunction (unadjusted OR 24.11 [7.95–73.05]); adjusted OR 23.16 [6.53–82.10]).  Incremental prognostic value of LV GLS was demonstrated (R 0.434→0.605, adjusted R^2^ 0.178 →0.355) | LV GLS cut-off value –19.9% to predict LV dysfunction with a sensitivity of 90% and a specificity of 79% (AUC 0.88 [0.83–0.93]; *p* < 0.001). |
| Pandis *et al.* [87], 2014, *J Am Soc Echocardiogr* | 130 | 57 ± 14 | 35 | severe/NA | Surgery (repair 100%) | NA | TomTec | Interobserver: ICC 0.83 (0.59–0.93);  Interobserver; variability 1.8%;  Intraobserver; variability 1.7% | 62.6 ± 11 | –20.4 ± 0.5 | Decrease in LVEF >10% | Immediate post-operatively | Better (higher magnitude) LV GLS on multivariate logistic regression was associated with LVEF reduction >10% after surgery (adjusted OR 0.81 [0.73–0.88]; *p* < 0.0001).  Incremental prognostic value of LV GLS was demonstrated (R^2^ 0.13→0.24, adjusted R^2^ 0.12 →0.23). | LV GLS cut-off value –20.5% to predict a post-operative decrease in LVEF >10% with a sensitivity of 66.7% and a specificity of 73% (AUC 0.75 [0.66–0.82]; *p* < 0.0001). |
|  |  |  |  |  |  |  |  |  |  |  | LVEF <50% in addition to LVEF reduction >10% | Immediate post-operatively | NA | LV GLS cut-off value –17.9% to predict LV dysfunction with a sensitivity of 80.7% and a specificity of 100% (AUC: 0.93 [0.84–0.98]; *p* < 0.0001). |
| Alashi *et al.* [80], 2016, *Circ Cardiovasc Imaging* | 448 | 61 ± 12 | 31 | moderate to severe or severe/asymptomatic | Surgery (repair 92%, replacement 8%) | Philips, Siemens, GE | Velocity Vector Imaging | Interobserver: ICC 0.86 (0.71–0.92), CV 7.4–8.7%;  Intraobserver: ICC 0.90 (0.81–0.97), CV 5.5–7.7% | 62 ± 3 | –20 ± 2 | All-cause mortality | 7.7 ± 2 years | Worse LV GLS on multivariable Cox survival analysis was associated with higher mortality (adjusted HR 1.17 [1.08–1.27], *p* < 0.002).  Incremental prognostic value of LV GLS was demonstrated (χ^2^ 47→61, *p* < 0.01; C-statistic 0.68→0.75, *p* < 0.01). | NA |
|  |  |  |  |  |  |  |  |  |  |  | LVEF <50% | 11 (10–12) months | Worse LV GLS on multivariate logistic regression was associated with post-operative LV dysfunction (adjusted OR 1.19 [1.09–1.30]). | NA |
| Cho *et al.* [81], 2016, *echocardiography* | 43 | 51.7 ± 14.3 | 42 | severe/both | Surgery (repair 79%, replacement 21%) | GE | EchoPAC | Interobserver: ICC 0.927 (0.846–0.965);  Intraobserver: ICC 0.942 (0.879–0.973) | 64.4 ± 6.5 | –20.3 ± 3.1 | LV remodeling (a decrease of GLS or LVEF that did not decrease LV end-diastolic dimension (LVEDD) | 3 months | Worse LV GLS on multivariate logistic regression was associated with post-operative LV remodeling or LV dysfunction (unadjusted OR 1.418 [1.083–1.856], adjusted OR 2.44 [1.259–4.729]). | LV GLS cut-off value –20.5% to predict LV remodeling with a sensitivity of 70% and a specificity of 75%. |
| Mentias *et al.* [82], 2016, *J Am Coll Cardiol* | 737 | 57.6 ± 13.4 | 32 | moderate to severe or severe/asymptomatic | surgery 65% (repair 92%, replacement 8%) + conservative (35%) | Philips, Siemens, GE | Velocity Vector Imaging | Interobserver: ICC 0.86 (0.71–0.92);  Intraobserver: ICC 0.89 (0.81–0.96) | 61.6 ± 2.1 | –21.6 ± 2.0 | All-cause mortality | 8.3 ± 3 years | For the subgroup after mitral surgery: better LV GLS than cut-off –21.7% (i.e., median value) on multivariable Cox survival analysis was associated with better survival (*p* < 0.001). | NA |
| Candan *et al.* [18], 2017, *Echocardiography* | 59 | 46.7 ± 13 | 46 | severe/asymptomatic | Surgery (repair 79%, replacement 21%) | GE | EchoPAC | NA | 65.2 ± 8 | –20.2 ± 4.5 | LVEF <50% | 8.3 ± 3 years | Worse LV GLS on univariate linear regression analysis was associated with poorer post-operative LVEF (r = 0.59; *p* < 0.001).  Worse LV GLS on multivariate logistic regression analysis was associated with post-operative LV dysfunction (adjusted OR 0.74 [0.56–0.98], *p* = 0.03). | LV GLS cut-off value –18.4% to predict LV dysfunction with a sensitivity of 85% and a specificity of 83% (AUC 0.844 [0.727–0.961]). |
| Kim *et al.* [39], 2018, *JACC Cardiovasc Imaging* | 506 | 58.5 ± 13.7 | 46 | severe/both | Surgery (repair 75%, replacement 25%) | NA | TomTec | Interobserver: ICC 0.96;  Intraobserver: NA | 59.9 ± 8.2 | –19.6 ± 4.2 | Cardiac events (cardiac death, worsening heart failure, reoperation for failure of MV surgery) | 3.5 (1.3–6.3) years | Worse LV GLS on multivariable Cox survival analysis was associated with higher cardiac events (adjusted HR 1.229 [1.135–1.331]).  Incremental prognostic value of LV GLS was demonstrated (χ^2^ 36.0→60.5 [*p* < 0.001]). | LV GLS cut-off –18.1% to predict cardiac events with a sensitivity of 71.4% and a specificity of 70.7% (AUC 0.738 [0.673–0.803]). |
|  |  |  |  |  |  |  |  |  |  |  | All-cause mortality | 3.5 (1.3–6.3) years | Worse LV GLS on multivariable Cox survival analysis was associated with higher mortality (adjusted HR 1.068 [1.003–1.136]). | NA |
| Hiemstra *et al.* [83], 2020, *JACC Cardiovasc Imaging* | 593 | 65 ± 12 | 36 | severe/both | Surgery (repair 98%, replacement 2%) | GE | EchoPAC | NA | 65 ± 8 | –21 ± 4 | All-cause mortality | 6.4 (3.6–10.4) years | Worse LV GLS on multivariable Cox survival analysis was associated with higher mortality (adjusted HR 1.13 [1.06–1.21]); better LV GLS than cutoff –20.6% (i.e., median value) was associated with better survival (log-rank *p* < 0.001). Better LV GLS than cutoff –19.5% and –22.3% was associated with better survival (log-rank *p* < 0.001).  Incremental prognostic value of LV GLS was demonstrated (C-statistic 0.74→0.77, *p* < 0.001) | NA |
|  |  |  |  |  |  |  |  |  |  |  | Cardiac events (cardiovascular death, heart failure hospitalizations, and cerebrovascular accidents) | 6.4 (3.6–10.4) years | Worse LV GLS on multivariable Cox survival analysis was associated with higher cardiac events (adjusted HR 1.08 [1.01–1.15], *p* = 0.019) | NA |
| Kislitsina *et al.* [84], 2020, *Ann Thorac Surg* | 119 (Strain was measured in 119/520 patients) | 58.5 ± 58.2 | 34 | ≥ moderate/both | Surgery (repair 98%, replacement 2%) | NA | TomTec | NA | Median 65 | –21.2 ± 4.2 | All-cause mortality | 5.0 ± 3.6 years | Baseline LV GLS on multivariable Cox survival analysis was not significantly associated with post-operative mortality (HR 0.932 [0.723–1.178], *p* = 0.573). | NA |
|  |  |  |  |  |  |  |  |  |  |  | LVEF <50% | Pre-discharge | Worse LV GLS on multivariate logistic regression was associated with post-operative LV dysfunction (OR 2.314 [1.528–3.504]).  Incremental prognostic value of strain (LV GLS, RV-GLS, LA-GLS) was demonstrated (AUC 0.687→0.955, *p* < 0.001). | NA |
| Stassen *et al.* [85], 2022, *Eur Heart J Cardiovasc Imaging* | 566 | 64 ± 12 | 34 | moderate to severe or severe/both | Surgery (repair 100%) | GE | EchoPAC | Interobserver: ICC –0.92 (0.84–0.97);  Intraobserver: ICC 0.94 (0.85–0.98) | 64 ± 8 | –21.4 ± 4.0 | All-cause mortality | 7 (4–12) years | Worse LV GLS on multivariable Cox survival analysis was associated with all-cause mortality (HR 0.924 [0.868–0.984], *p* = 0.014). | NA |
| Althunayyan *et al.* [24], 2023, *Eur Heart J Cardiovasc Imaging* | 87 | 64 (54–72) | 40 | severe/both | Surgery (repair 77%, replacement 23%) | GE | EchoPAC | NA | 62 (58–66) | –20 ± 3 | LVEF <50% | 12 months | Worse LV GLS on multivariate logistic regression was associated with post-operative LV dysfunction (OR 1.46 [1.00–2.14], *p* = 0.054). | LV GLS cut-off –19.5% to predict LV dysfunction with a sensitivity of 73% and a specificity of 68% (AUC 0.738 [0.56–0.71). |

The Cell colors conform with Fig. 1. LV GLS, left ventricular global longitudinal strain; LV, left ventricle; LVEF, left ventricular ejection fraction; ICC, intraclass correlation coefficients; CV, coefficient of variation; NA, not available; AUC, area under curve; OR, odds ratio; HR, hazard ratio.

Supplementary Table 8. Description of included studies of left atrial strain.

| First Author [Ref.]; Year Published; *Journal* | Total Number of Participants | Age | Female (%) | Population (severity/symptom) | Surgery | Vendor for echocardiography | Software for strain analysis | Reproducibility (interobserver/intraobserver) | Baseline LVEF (%) | Baseline LA reservoir strain (%) | Primary Outcome and Composite Cardiac Endpoint | Follow-up | Association Between LA Strain and Outcomes | Receiver-Operating Characteristic Curve Analysis |
| --- | --- | --- | --- | --- | --- | --- | --- | --- | --- | --- | --- | --- | --- | --- |
| Debonnaire *et al.* [89], 2013, *J Am Soc Echocardiogr* | 121 | 63 ± 13 | 36 | severe/both | surgery 96.7% + conservative (3.3%) | GE | EchoPAC | Interobserver: ICC 1.1% (–4.2% to 6.4%);  Intraobserver: ICC –1.3% (–6.6% to 3.9%) | 68 ± 8 | 22 ± 8.4 | All-cause mortality | 6.4 (4.7–8.7) years | Worse LA reservoir strain on multivariable Cox survival analysis was significantly associated with mortality (HR 3.5 [1.03–12.20], *p* = 0.045)  The incremental prognostic value of LA reservoir strain was demonstrated by the likelihood ratio test (*p* = 0.03). | NA |
| Candan *et al.* [42], 2014, *Int J Cardiovasc Imaging* | 53 | 45.7 ± 13.5 | 55 | severe/both | Surgery (repair 56.6%, replacement 43.4%) | GE | EchoPAC | Interobserver: absolute bias 2.7 (–1.9 to 7.3);  Intraobserver: absolute bias 0.90 (0.84–0.95). | 64.6 ± 7.1 | NA | LARR (Reduction of indexed LA Volume) | >6 months | Better LA reservoir strain on multivariate linear regression analysis was associated with better LA reverse remodeling (β = 0.54, *p* = 0.001). | NA |
| Kislitsina *et al.* [84], 2020, *Ann Thorac Surg* | 119 (Strain was measured in 119/520 patients) | 58.5 ± 58.2 | 34 | ≥ moderate/both | Surgery (repair 98%, replacement 2%) | NA | TomTec | NA | Median 65 | –19.9 ± 4.2% | All-cause mortality | 5.0 ± 3.6 years | Baseline LA reservoir strain on multivariate Cox survival analysis was not significantly associated with post-operative mortality (HR 0.931 [0.855–1.016], *p* = 0.931). | NA |
|  |  |  |  |  |  |  |  |  |  |  | LVEF <50% | Pre-discharge | Worse LA reservoir strain on multivariate logistic regression was associated with post-operative LV dysfunction (OR 0.888 [0.808–0.977]).  Incremental prognostic value of strain (LV GLS, RV-GLS, LA reservoir strain) was demonstrated (AUC 0.687→0.955, *p* < 0.001) | NA |
| Mandoli *et al.* [90], 2021, *Int J Cardiol* | 65 | 71 ± 8 | 39 | severe/both | Surgery (repair 69%, replacement 31%) | GE | EchoPAC | NA | 60.1 ± 1.0 | 22.9 ± 5.5 | Composite events (Heart failure and mortality) | 3.7 ± 1 years for the event-group, 6.8 ± 1 years for non-event group | Worse LA reservoir strain on multivariate Cox survival models was all significantly associated with higher mortality (HR 0.74–0.91, all the *p* < 0.05).  The incremental prognostic value of strain LA reservoir strain was demonstrated (χ^2^ 6.80→17.61, *p* = 0.001). | LA reservoir strain cut-off value –21% to predict post-operative composite events (AUC 0.78, *p* < 0.01). |
|  |  |  |  |  |  |  |  |  |  |  | Post-operative functional capacity (NYHA class and Borg CR 10 scale) | 3.7 ± 1 years for the event-group, 6.8 ± 1 years for non-event group | LA reservoir strain was associated with post-operative NYHA class (r^2^ = 0.11, *p* = 0.04) and Borg CR 10 scale (r^2^ = 0.10, *p* = 0.02). | NA |
| Stassen *et al.* [85], 2022, *Eur Heart J Cardiovasc Imaging* | 566 | 64 ± 12 | 34 | moderate to severe or severe/both | Surgery (repair 100%) | GE | EchoPAC | Interobserver: ICC 0.92 (0.84–0.97);  Intraobserver: ICC 0.94 (0.85–0.98) | 64 ± 8 | 23.0 ± 9.7 | All-cause mortality | 7 (4–12) years | Worse LA reservoir strain on multivariable Cox survival analysis was significantly associated with higher all-cause mortality (HR 0.961 [0.932–0.992]; *p* = 0.014).  The incremental prognostic value of LA reservoir strain was demonstrated (χ^2^ 98.3→105.2, *p* = 0.011). | LA reservoir strain PALS cut-off value –22% derived from spline curve to predict all-cause mortality (i.e., in which the predicted HR is ≥1). |

The Cell colors conform with Fig. 1. LA, left atrium; LV, left ventricle; LVEF, left ventricular ejection fraction; ICC, intraclass correlation coefficients; CV, coefficient of variation; NA, not available; AUC, area under curve; LV GLS, left ventricular global longitudinal strain; OR, odds ratio; HR, hazard ratio.

Supplementary Table 9. Description of included studies of stress echocardiography.

| First Author [Ref.]; Year Published; *Journal* | Total Number of Participants | Age | Female (%) | Population (severity/symptom) | Surgery | Reproducibility (interobserver/intraobserver) | Baseline LVEF (%) | Baseline stress-induced parameters (mm) | Outcomes | Follow-up | Association Between Stress Echocardiography and Outcomes | Receiver-Operating Characteristic Curve Analysis |
| --- | --- | --- | --- | --- | --- | --- | --- | --- | --- | --- | --- | --- |
| Leung *et al.* [96], 1996, *J Am Coll Cardiol* | 74 | 54 ± 13 | 26 | moderate and severe/asymptomatic | Surgery (repair 100%) | Interobserver: CV and ICC for LVEDD, LVESD, and LVEF after exercise were 1.2 ± 14.5 cm^3^（r = 0.94), –1.8 ± 4.7 cm^3^ (r = 0.96), and 1 ± 3 cm^3^, respectively.  Intraobserver: CV and ICC for LVEDD, LVESD, and LVEF after exercise were –0.8 ± 9.5 cm^3^ (r = 0.98), –0.9 ± 3.2 cm^3^ (r = 0.99), and –1 ± 2 cm^3^. respectively. | 64 ± 9 | LVEDV_ex_: 135 ± 32 cm^3^;  LVESV_ex_: 41 ± 17 cm^3^;  LVESVi_ex_: 21 ± 9 cm^3^;  LVEF_ex_: 70 ± 12%  LVEDV_rest_: 145 ± 39 cm^3^;  LVESV_rest_: 52 ± 20 cm^3^;  LVESVi_rest_: 27 ± 10 cm^3^;  LVEF_rest_: 64 ± 9% | LVEF <50% | 8 ± 14 days | Smaller LVEF_ex_ (*p* = 0.03) and Larger LVESVi_ex_ (*p* = 0.003) on the multivariate linear regression model were associated with poorer post-operative LVEF (multiple R = 0.78, *p* < 0.0005) | LVESVi_ex_ cut-off value 25 cm^3^/m^2^ to predict post-operative LV dysfunction with a sensitivity of 83% and specificity of 83%.  LVEF_ex_ cut-off value 68% to predict post-operative LV dysfunction with a sensitivity of 80% and specificity of 81%. |
| Lee *et al.* [99], 2005, *Heart* | 71 | 61 ± 3 | 32 | severe/asymptomatic | surgery (repair 75.0%, replacement 25.0%) | Interobserver: CV 4% (rest), 2% (exercise);  Intraobserver: NA | 64 ± 7 | Δ LVEF: 3.4 ± 8.7% | LVEF <50% | 3 ± 1 years | For the subgroup after mitral surgery:  Lower Δ LVEF on multivariate linear regression model was associated with poorer post-operative LVEF (*p* = 0.006). | NA |
| Lancellotti *et al.* [95], 2008, *J Am Soc Echocardiogr* | 30 | 64 ± 13 | 40 | severe/asymptomatic | surgery (repair 76.7%, replacement 23.3%) | Interobserver: ICC 0.86 (rest), 0.91 (exercise);  Intraobserver: ICC 0.89 (rest), 0.87 (exercise) | 67 ± 6 | Δ LV GLS: 1.7 ± 4.4% | LVEF <50% | 13 ± 8.8 months (1.1 ± 0.7 years) | Lower Δ LV GLS on multivariate linear regression model was associated with poorer post-operative LVEF (*p* = 0.001). | Δ LV GLS cut-off value 1.9% to predict post-operative LV dysfunction with a sensitivity of 92.3% and specificity of 70.6%. |
| Donal *et al.* [86], 2012, *Eur Heart J Cardiovasc Imaging* | 77 | 63 ± 16 | 33 | severe/asymptomatic | surgery (repair 87.5%, replacement 12.5%) | Interobserver: CV 13.5% (rest), 6.1% (exercise);  Intraobserver: NA | 67 ± 12 | LV GLS_ex_: 21 ± 6% | LVEF <50% | 6 ± 1 months | Worse LV GLS_ex_ normalized for LVESD on a multivariate linear regression model was associated with poorer post-operative LVEF (*p* = 0.019). | LVGLS_ex_ normalized for LVESD cut-off value –5.7%/cm to predict post-operative LV dysfunction with a sensitivity of 83% and specificity of 70%. |
| Magne *et al.* [94], 2015, *Heart* | 102 | 64 ± 12 | 32 | moderate to severe/asymptomatic or mildly symptomatic | surgery (repair 78%, replacement 22%) | NA | 71 (66, 76) | SPAP_ex_: (63 ± 18) mmHg; | Major adverse cardiovascular events (occurrence of atrial fibrillation, stroke, cardiac-related hospitalization or death) | 50 ± 23 months (4.2 ± 1.9 years) | PH_ex_ (>60 mmHg) on multivariate Cox survival analysis was associated with more frequent post-operative major adverse cardiovascular events in all models (*p* ≤ 0.04). | PH_ex_ was defined as an SPSP >60 mmHg at exercise as recommended by current guidelines. |
| Vitel *et al.* [93], 2018, *Heart* | 142 | 58 ± 21 | 32 | severe/asymptomatic | Surgery (repair 85.9%, replacement 14.1%) | NA | 66 ± 6 | LVEF_ex_: (70 ± 6.8)%; ΔLVEF: (3.2 ± 7.5)%;  LV GLS_ex_: (–22 ± 4.1)%; ΔLV GLS: (–1.9 ± 3.6)%;  TAPSE_ex_: (26 ± 5.8) mm; ΔTAPSE: (3 ± 5.7) mm;  S'_ex_: (17 ± 4) cm/s; ΔS': (3.44 ± 3.93) cm/s;  RV FAC_ex_: (47 ± 9.3)%; ΔRV FAC: (2.2 ± 10)%;  RV GLS_ex_: (–26 ± 7.3)%; ΔRV GLS: (–0.96 ± 5.5)%;  SPAP_ex_: (58 ± 20) mmHg; ΔsPAP: (22 ± 15) mmHg. | Major adverse cardiovascular events (occurrence of atrial fibrillation, stroke, cardiac-related hospitalization or death) | 30 (16–60) months (2.5 (1.3, 5.0) years) | TAPSE_ex_ on multivariate Cox survival analysis (Bayesian model averaging method) was associated with post-operative major adverse cardiovascular events (HR 0.91 [0.86–0.96], PrP = 74%). | TPASE_ex_ cut-off value 26 mm to predict major adverse cardiovascular events. (AUC 0.83 [0.73–0.91]). |

The Cell colors conform with Fig. 1. Δ, difference between exercise and rest; _ex_, excise; LVEF, left ventricular ejection fraction; LVEDD, left ventricular end-diastolic diameter; LVESD, left ventricular end-systolic diameter; LVEDV, left ventricular end-diastolic volume; LVESV, left ventricular end-systolic volume; LVESVi, left ventricular end-systolic volume index; LV GLS, left ventricular global longitudinal strain; SPAP, systolic pulmonary artery pressure; PH, pulmonary hypertension; TAPSE, tricuspid annular plane systolic excursion; RV FAC, right ventricular fractional area change; RV GLS, right ventricular global longitudinal strain; ICC, intraclass correlation coefficients; CV, coefficient of variation; NA, not available; AUC, area under curve; OR, odds ratio; HR, hazard ratio.
